# PucC and LhaA direct efficient assembly of the light‐harvesting complexes in *Rhodobacter sphaeroides*

**DOI:** 10.1111/mmi.13235

**Published:** 2015-11-05

**Authors:** David J. Mothersole, Philip J. Jackson, Cvetelin Vasilev, Jaimey D. Tucker, Amanda A. Brindley, Mark J. Dickman, C. Neil Hunter

**Affiliations:** ^1^ Department of Molecular Biology and BiotechnologyUniversity of SheffieldFirth Court, Western BankSheffieldS10 2TNUK; ^2^ ChELSI Institute, Department of Chemical and Biological EngineeringUniversity of SheffieldMappin StreetSheffieldS1 3JDUK; ^3^ Present address: Section for Marine BiologyDepartment of BiologyUniversity of CopenhagenStrandpromenaden 53000HelsingørDenmark

## Abstract

The mature architecture of the photosynthetic membrane of the purple phototroph *R* *hodobacter sphaeroides* has been characterised to a level where an atomic‐level membrane model is available, but the roles of the putative assembly proteins LhaA and PucC in establishing this architecture are unknown. Here we investigate the assembly of light‐harvesting LH2 and reaction centre‐light‐harvesting1‐PufX (RC‐LH1‐PufX) photosystem complexes using spectroscopy, pull‐downs, native gel electrophoresis, quantitative mass spectrometry and fluorescence lifetime microscopy to characterise a series of *lha* *A* and *puc* *C* mutants. LhaA and PucC are important for specific assembly of LH1 or LH2 complexes, respectively, but they are not essential; the few LH1 subunits found in Δ*lha* *A* mutants assemble to form normal RC‐LH1‐PufX core complexes showing that, once initiated, LH1 assembly round the RC is cooperative and proceeds to completion. LhaA and PucC form oligomers at sites of initiation of membrane invagination; LhaA associates with RCs, bacteriochlorophyll synthase (BchG), the protein translocase subunit YajC and the YidC membrane protein insertase. These associations within membrane nanodomains likely maximise interactions between pigments newly arriving from BchG and nascent proteins within the SecYEG‐SecDF‐YajC‐YidC assembly machinery, thereby co‐ordinating pigment delivery, the co‐translational insertion of LH polypeptides and their folding and assembly to form photosynthetic complexes.

## Introduction

The purple phototroph *Rhodobacter (Rba.) sphaeroides* houses an extensive system of vesicular intracytoplasmic membranes, which increases the internal surface area of membranes and photosystem complexes for absorbing and trapping solar energy. Energy harvested by light‐harvesting LH2 complexes migrates to the reaction centre‐light‐harvesting 1‐PufX (RC‐LH1‐PufX) complex where charge separations drive the reduction of quinones to quinols, which diffuse to nearby cytochrome *bc*_1_ complexes (Lavergne *et al*., 2008; Cartron *et al*., 2014). Here, the quinols are oxidised, and the resulting proton‐motive force is used for the production of ATP and also for other energy‐consuming processes such as motility. The architecture of the *Rba. sphaeroides* photosynthetic membrane has been highly characterised by atomic force microscopy, spectroscopic techniques and tomographic imaging (Bahatyrova *et al*., 2004a; Frese *et al*., 2004; Sturgis *et al*., 2009; Tucker *et al*., 2010; Adams and Hunter, 2012), which has culminated in a 1.9 million‐atom structural model of the photosynthetic vesicle, or chromatophore (Cartron *et al*., 2014). Despite the importance and usefulness of purple bacterial LH and RC complexes as models for studying the light‐driven reactions of photosynthesis, little is known about the proteins that govern their assembly.

**Figure 1:**
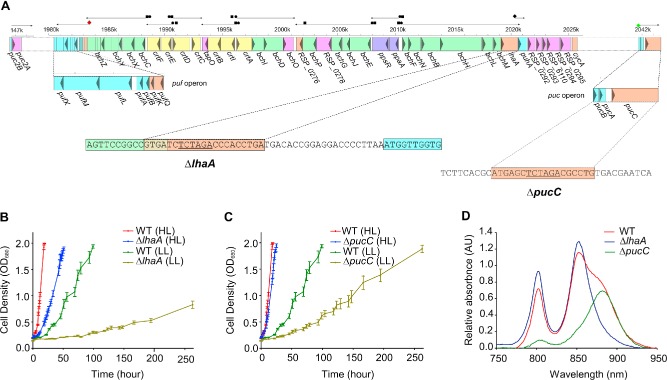
Photosynthetic growth of Δ*lha* *A* and Δ*puc* *C* mutants. A. Schematic of the photosynthesis gene cluster of *R* *ba. sphaeroides*, adapted from Naylor *et al*. (1999). Map numbers correspond to the base pair number in thousands. Arrows above map numbers indicate genes grouped into operons within the cluster. Arrow heads within genes indicate direction of transcription. Above the genes black circles indicate palindromic regulatory elements, black squares indicate possible *E* *. coli* σ‐like promoter, circles and squares together indicate overlapping palindrome and *E* *. coli* σ‐like promoter sequences, the red diamond is the *puf* promoter and the black diamond is a *puf*‐like promoter. The green diamond is the *puc* promoter. The expanded sequences show the locations of the modified genome sequences for Δ*lha* *A* and Δ*puc* *C* mutants. B. Photosynthetic growth curves for the Δ*lha* *A* and wild‐type strains at high (HL; 100 μmol photons s^−1^ m^−2^) and low (LL; 10 μmol photons s^−1^ m^−2^) light intensities. Error bars are standard error and *n* = 3. Three biological replicates were measured for each time point. C. Photosynthetic growth curves for the Δ*puc* *C* and wild‐type strains at high and low light intensities, as in B. D. Whole cell absorbance spectra of Δ*lha* *A* (blue), Δ*puc* *C* (green) and wild‐type (red) strains normalised to cell density (absorbance at 680 nm) and baselined with a high‐oxygen wild‐type culture containing no pigments. **This figure is available in colour online at** wileyonlinelibrary.com.

In photosynthetic bacteria such as *Rba. capsulatus* and *Rba. sphaeroides* most of the genes that encode the pigment biosynthetic pathways, putative assembly factors and the photosystem apoproteins are located within a photosynthesis gene cluster (PGC) (Fig. 1A). The *Rba. sphaeroides* PGC includes genes involved in the carotenoid and bacteriochlorophyll (BChl) biosynthesis pathways and in regulating responses to oxygen and light, which are sandwiched between genes encoding apoproteins of the RC‐LH1‐PufX core complex (Naylor *et al*., 1999; see Fig. 1A). The *puf* operon encodes the LH1 α, β polypeptides, the RC‐L, M subunits and the PufX polypeptide; *puhA*, encoding the remaining RC subunit, RC‐H, is located 45 kb from the *puf* operon (Coomber *et al*., 1990; Naylor *et al*., 1999). Structural genes required for the biosynthesis of LH2 are not located within the PGC, and instead they are present in two separate operons located in different areas of the *Rba. sphaeroides* genome (Zeng *et al*., 2003). The *puc1BAC* operon contains open reading frames encoding the LH2 α and β polypeptides (Ashby *et al*., 1987; Kiley and Kaplan, 1987), which form a nonameric ring of LH2 αβ‐heterodimers (Walz *et al*., 1998).

The first indications of the existence of photosystem assembly factors came from early sequencing and insertion mutagenesis experiments with *Rba. capsulatus* that identified *F1696* encoding a 477‐amino acid hydrophobic protein (Zsebo and Hearst, 1984). Transposon insertions into *F1696*, now *lhaA*, reduced LH1 levels and polar effects on RC assembly, likely affecting *puhA* encoding the RC‐H subunit, were also noted. Interposon mutagenesis of *F1696* produced similar effects, and complementation with a plasmid containing *F1696* restored LH1 to WT levels (Young *et al*., 1998). With regard to LH2 assembly *pucC* lies downstream of *puc1BA* encoding the main LH2 β and α polypeptides, sharing the same promoter; interruption of *pucC* greatly reduces assembly of LH2 complexes in *Rba. capsulatus*, *Rba. sphaeroides* and *Rubrivivax gelatinosus* (Donohue *et al*., 1988; Tichy *et al*., 1989; Gibson *et al*., 1992; LeBlanc and Beatty, 1993; Steunou *et al*., 2004). On the basis of PhoA translational fusions Beatty and co‐workers proposed topological models of the *Rba. capsulatus* LhaA and PucC proteins, each consisting of 12 transmembrane helices with the N‐ and C‐termini located in the cytoplasm (LeBlanc and Beatty, 1996; Young and Beatty, 1998). Topological models predict a similar arrangement for the transmembrane helices of PucC (Simmons *et al*., 2000). LhaA and PucC proteins have been assigned as members of a family of bacteriochlorophyll delivery proteins, within the major facilitator superfamily of transmembrane solute transporters (Saier *et al*., 1999).

Here, we have constructed genomic deletions of *lhaA* and *pucC* to circumvent problems with insertional mutagenesis. Measurement of photosynthetic growth rates, the spectroscopic properties of these mutants, and analysis by native gels, quantitative mass spectrometry and lifetime microscopy has provided new insights into the assembly of this model bacterial photosystem. We show that although LhaA and PucC are important for assembly of LH1 or LH2 complexes, respectively, they are not essential. The few LH1 subunits made in the absence of LhaA form native RC‐LH1‐PufX monomer and dimer complexes, leaving many ‘free’ RCs with no encircling LH1 and showing that, once initiated, LH1 assembly round the RC is cooperative and proceeds to completion. LhaA and PucC are found preferentially at sites of initiation of membrane invagination, in an oligomeric state and with LhaA in association with RCs as well as enzymes involved in pigment biosynthesis and membrane protein insertion.

## Results

### Construction, growth rates, absorption spectroscopy and biochemical analysis of ΔlhaA and ΔpucC mutants

The *lhaA* and *pucC* deletion constructs containing the upstream and downstream flanking regions of each gene (Fig. 1A) were created using primers displayed in Table S1. Instead of completely deleting the genes 18 bp was left in place in the Δ*lhaA* construct to prevent disruption of the overlapping *bchM* (Fig. 1A) that encodes magnesium protoporphyrin monomethyltransferase, an enzyme early in the BChl biosynthesis pathway (Gibson and Hunter, 1994). Conjugation of the constructs, verified by sequencing, was performed as described in *Experimental procedures* using the WT, Δ*pucC* and Δ*pucBA Δpuc2BA* strains as recipients.

Figure 1B shows that the Δ*lhaA* mutant grows more slowly than the wild‐type under high light (1000 μmol photons s^−1^ m^−2^) conditions, and the rate of photosynthetic growth is severely impaired under low light (10 μmol photons s^−1^ m^−2^) (Fig. 1B; blue and red curves). The retention of some photosynthetic growth is an indication that LhaA is not involved in assembly of the RC. Similar results were obtained for Δ*pucC* (Fig. 1C), albeit with less effect on the growth rates. Whole cell absorption spectra (Fig. 1D) show the loss of the LH1 ‘shoulder’ at 875 nm in the Δ*lhaA* mutant, which leaves the 800 and 850 nm absorption bands characteristic of the LH2 complex; conversely the Δ*pucC* mutant lacks these LH2 absorption bands and retains the LH1 absorption band at 875 nm, as well as a small feature near 800 nm from the RC.

**Figure 2:**
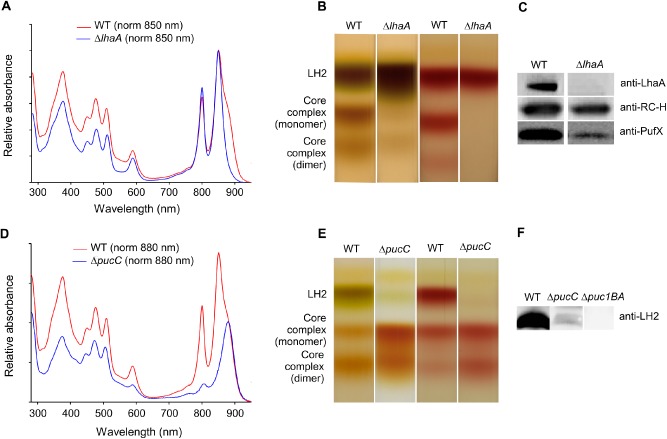
Analyses of intracytoplasmic membranes from *lha* *A* and *puc* *C* deletion mutants by spectroscopy, detergent fractionation of complexes and immunoblotting. A. Room temperature absorbance spectra of membranes prepared from WT (red) and *Δlha* *A* (blue) strains normalised to LH2 absorbance at 850 nm. B. Fractionation of detergent‐solubilised photosystem complexes from WT and Δ*lha* *A* strains on sucrose density gradients. (Left pair of gradients) complexes from cells grown under photosynthetic conditions. (Right) Complexes from cells grown under semi aerobic conditions. C. Immunoblots of membranes from photosynthetically grown WT and *Δlha* *A* strains probed by antibodies to LhaA, RC‐H and PufX polypeptides. The sample loadings were normalised to LH2 absorbance at 850 nm. D. Room temperature absorbance spectra of membranes prepared from WT (red) and *Δpuc* *C* (blue) strains normalised to LH1 absorbance at 880 nm. E. Fractionation of detergent‐solubilised photosystem complexes from WT and Δ*puc* *C* strains on sucrose density gradients. (Left pair of gradients) Complexes from cells grown under photosynthetic conditions. (Right) Complexes from cells grown under semi‐aerobic conditions. F. Immunoblots of membranes from photosynthetically grown WT, *Δpuc* *C* and *Δpuc1* *BA* strains probed by antibodies to LH2 polypeptides. The sample loadings were normalised to LH1 absorbance at 875 nm. **This figure is available in colour online at** wileyonlinelibrary.com.

In order to investigate the effects of the *lhaA* and *pucC* deletions in more detail, membranes were prepared from photosynthetically grown cultures. The room temperature absorbance spectrum of the Δ*lhaA* mutant (Fig. 2A, blue curve) confirmed the near absence of LH1 complex absorption at 875 nm relative to the WT (Fig. 2A, red curve). Discontinuous sucrose density gradients of WT and *ΔlhaA* membranes solubilised with 3% n‐dodecyl‐β‐D‐maltoside (β‐DDM) and fractionated as described in *Experimental procedures* (Fig. 2B) demonstrated that the strain is still capable of producing monomeric and dimeric RC‐LH1‐PufX core complexes, albeit at very low levels. This observation holds for membranes prepared from anaerobic, photosynthetically grown cells, but membranes prepared from cells grown under oxygen‐limited conditions in the dark contained undetectably low levels of these complexes (Fig. 2B; right‐hand pair of gradients). Immunoblotting (Fig. 2C) confirmed the absence of LhaA from the Δ*lhaA* mutant; retention of the RC‐H subunit immunoblot signal shows that deletion of *lhaA* does not affect *puhA*, which lies immediately downstream of *lhaA* (see Fig. 1A). The immunoblot of the *ΔlhaA* strain shows reduced levels of the PufX polypeptide, a crucial component of the core complex responsible for enabling quinone/quinol exchange across the LH1 ring encircling the RC (Farchaus *et al*., 1990; Lilburn *et al*., 1992; McGlynn *et al*., 1994; 1996).

Room temperature spectra (Fig. 2D) of membranes prepared from the Δ*pucC* mutant demonstrated the apparent absence of the LH2 800 and 850 nm absorption peaks. However, discontinuous sucrose density gradients of WT and Δ*pucC* membranes solubilised in 3% β‐DDM showed that the Δ*pucC* strain still assembles very low levels of LH2 (Fig. 2E), consistent with the weak immunoblotting signal from the LH2 α and β polypeptides in this mutant (Fig. 2F). Taken together, the analyses in Fig. 2 illustrate the specificity of LhaA and PucC for LH1 and LH2 assembly, respectively, but also show that low levels of these antenna complexes could be detected despite deletion of *lhaA* or *pucC*.

### Analysis of a ΔlhaA ΔpucC double deletion strain

**Figure 3:**
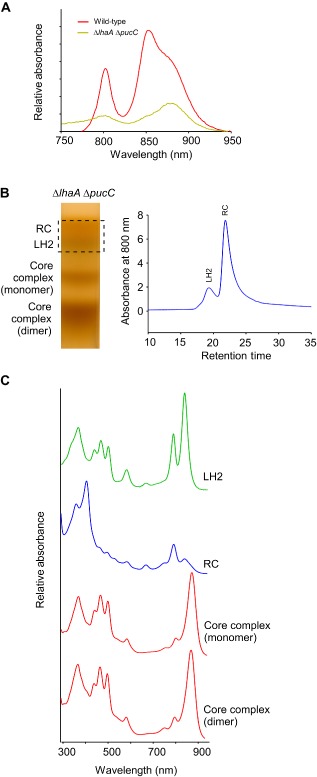
Phenotype of a mutant with deletions in both *lha* *A* and *puc* *C*. A. Absorbance spectra of membranes from wild‐type (red) and Δ*lha* *A* Δ*puc* *C* (green) strains, prepared from photosynthetically grown cells. The spectra were normalised for the protein content of the samples. B. Fractionation of detergent‐solubilised photosystem complexes from the Δ*lha* *A* Δ*puc* *C* mutant on (left) a sucrose density gradient, then (right) separation of LH2 and RC complexes by HPLC gel filtration. C. Absorbance spectra of the four separate complexes identified in (B). **This figure is available in colour online at** wileyonlinelibrary.com.

The analyses of single *ΔlhaA* or *ΔpucC* deletions, each containing some residual LH1 or LH2 complex, respectively, could be explained by some ‘crosstalk’, with PucC permitting some LH1 assembly in the *ΔlhaA* strain, and LhaA permitting some LH2 assembly in the *ΔpucC* mutant. In order to investigate this point further, we constructed a *ΔlhaA ΔpucC* double deletion strain which, despite loss of the majority of photosystem complexes, still grew photosynthetically (results not shown). The whole cell absorption spectrum of the *ΔlhaA ΔpucC* strain (Fig. 3A) shows that LH2 levels are particularly affected and although the dominant absorption in the WT is the 850 nm LH2 peak (Fig. 3A, red spectrum), the major absorption feature in cells and membranes of this double deletion strain arises from LH1, at 875 nm. Despite the low levels of complexes in *ΔlhaA ΔpucC* membranes, it was possible to solubilise them in detergent and, with a high sample loading, fractionate them on sucrose density gradients (Fig. 3B, left) although a subsequent high‐performance liquid chromatography (HPLC) gel filtration step (Fig. 3B, right) was required to separate the two uppermost bands in the gradient and identify them individually as LH2 and RC complexes (Fig. 3C). Thus, four complexes, identified by their absorption spectra (Fig. 3C), were present in membranes of the *ΔlhaA ΔpucC* strain: the LH2 complex, RCs free of any encircling LH1 complexes, and monomeric and dimeric core complexes with apparently normal LH1 composition. This result underlines the ‘leaky’ nature of the *lhaA* and *pucC* deletions, which still permit low levels of LH complex assembly, and it also hints at an intriguing aspect of RC‐LH1‐PufX assembly that favours complete encirclement of RCs even at the expense of leaving some RCs with no LH1 attached. This point is examined further below.

### Analysis of ΔlhaA in an LH2^−^ background

**Figure 4:**
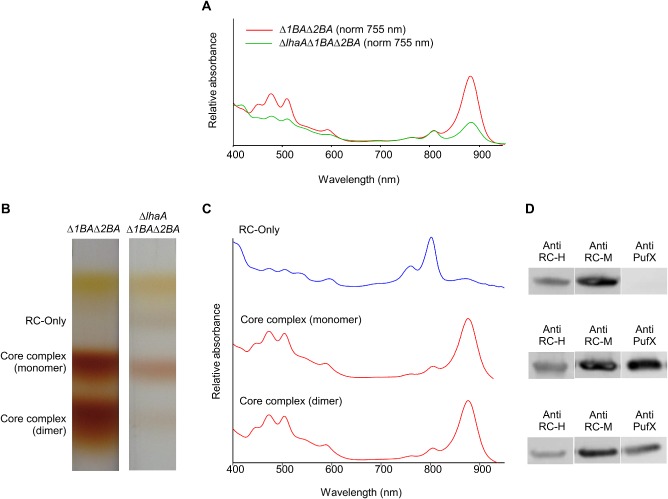
Assembly of some normal core complexes despite the deletion of *lha* *A*. A. Absorbance spectrum of membranes from an LH2^−^ (Δ*puc1* *BA* Δ*puc2* *BA*) strain, showing RC‐LH1‐PufX complexes (red), and membranes from the Δ*lha* *A* Δ*puc1* *BA* Δ*puc2* *BA* strain (green). The spectra were normalised to RC absorption at 755 nm. B. Fractionation of detergent‐solubilised complexes from Δ*puc1* *BA* *Δpuc2* *BA* and Δ*puc1* *BA* Δ*puc2* *BA* *Δlha* *A* on sucrose density gradients, loaded with equal amounts of total protein. C. Absorbance spectra of bands harvested from the Δ*puc1* *BA* Δ*puc2* *BA* Δ*lha* *A* gradient. D. Immunoblots of the three complexes in (C), probed with antibodies to the RC‐H, ‐M and PufX polypeptides, with sample loadings normalised to the reaction centre 800 nm absorbance peak. **This figure is available in colour online at** wileyonlinelibrary.com.

In order to observe the effects of the *lhaA* deletion without any obscuring effects of the LH2 complex, the Δ*lhaA* mutation was introduced into an LH2^−^ (Δ*puc1BA* Δ*puc2BA*) strain constructed by deleting both sets of genes encoding LH2 α and β polypeptides. The Δ*lhaA* Δ*puc1BA* Δ*puc2BA* and Δ*puc1BA* Δ*puc2BA* strains were both grown photosynthetically; Fig. 4A displays absorption spectra of membranes prepared from these two strains, normalised to RC absorption at 800 nm. The effect of deleting *lhaA* is clear; the LH1 absorbance band at 875 nm is significantly reduced but not abolished, as also seen with the *lhaA* deletion in the WT background in Fig. 2. Analysis of detergent‐solubilised membranes on discontinuous sucrose density gradients (Fig. 4B) showed an uppermost orange band containing carotenoids; below the carotenoid band there are the expected monomeric and dimeric RC‐LH1‐PufX cores in the control sample, whereas levels of these complexes are much reduced in the *ΔlhaA* mutant and a new RC‐only band has appeared, reflecting the lack of LH1 subunits available to encircle the RC. Analysis of the 800 nm RC absorbance of the pigmented bands recovered from each gradient (not shown) demonstrates that approximately 85% of the RCs in the Δ*lhaA* mutant are in the LH1‐free state. Fig. 4C (top) shows the absorbance spectrum for the uppermost pigment‐protein band recovered from the Δ*lhaA* Δ*puc1BA* Δ*puc2BA* gradient, confirming the presence of RC‐only complexes; the spectra of the two lower bands, arising from RC‐LH1‐PufX core monomer and dimers (Crouch *et al*., 2010; Ratcliffe *et al*., 2011), are apparently unaffected by the much‐reduced levels of LH1 caused by deleting *lhaA*. These spectra of the core complexes retrieved from the Δ*lhaA* Δ*puc1BA* Δ*puc2BA* strain were compared with a control spectrum recorded on the intact RC‐LH1‐PufX complex prepared from the Δ*puc1BA* Δ*puc2BA* (that is, LH2^−^ minus) strain. The ratios of LH1 (875 nm): RC (802 nm) absorption for the core monomer and core dimer spectra are 5.2 and 5.0 respectively; control absorption spectra for detergent‐solubilised complexes containing the same major carotenoid, spheroidene (Chi *et al*., 2015), are 4.8 and 5.3 showing that the WT LH1:RC stoichiometry, characteristic of an intact core complex with its full complement of encircling LH1, has been largely maintained in the few core complexes that do assemble, despite the loss of most of the LH1 complex from the membrane due to the *lhaA* deletion. This is a significant result and it shows that, once initiated, assembly of LH1 subunits round the RC proceeds to completion, even at the expense of leaving other RCs with no associated LH1 subunits, shown by the substantial RC‐only fraction in Fig. 4B (right). Immunoblotting of the RC‐only and core complex bands (Fig. 4D), with samples normalised to the reaction centre 800 nm peak, verifies the expected presence of the reaction centre H and M subunits, and also shows that the PufX polypeptide is absent from the RC‐only band.

### Analysis of RC‐LH1‐PufX complex polypeptides in the ΔlhaA mutant by quantitative mass spectrometry

In order to quantify the effects of the Δ*lhaA* mutation on RC‐LH1‐PufX polypeptides, membranes were prepared from Δ*lhaA* and WT strains grown under photosynthetic conditions, as described in *Experimental procedures*. An ^15^N‐labelled artificial protein was constructed for use as an internal standard, composed of concatenated tryptic peptide sequences known to represent RC‐M, RC‐L, LH1 α and PufX (Table S2) target proteins, and used for their quantification in previous proteomic analyses (Qian *et al*., 2013; Cartron *et al*., 2014; Olsen *et al*., 2014). Table 1 shows the quantities of tryptic peptides corresponding to the RC‐M, RC‐L, LH1 α and PufX polypeptides; the data were normalised to the RC‐M1 tryptic peptide (Table S2) to allow a quantitative comparison of the LH1 α and PufX levels. Table 1 shows that RC‐M and RC‐L are present in an approximate 1:1 stoichiometry, consistent with the structure of the RC complex (Allen *et al*., 1987), but the LH1 α : RC‐M or RC‐L ratios of 14:1 expected from the RC‐LH1‐PuX structure (Qian *et al*., 2013) were not seen, and instead a lower ratio of 8:1 was observed (Table 1). This discrepancy is likely a consequence of incomplete trypsin digestion of the LH1 α polypeptide, but our protocol nevertheless enabled relative quantification, clearly showing that LH1 α and PufX levels have fallen by 87% and 61% respectively in the Δ*lhaA* mutant, relative to the RC, reflecting the qualitative conclusions drawn from the sucrose density gradients in Fig. 4B.

**Table 1:** Quantification of RC‐LH1‐PufX complex polypeptides in wild‐type and *Δlha* *A* strains by mass spectrometry

Target protein	Tryptic peptide	Quantity in WT (μmol g^−1^ total protein)	Stoichiometry in WT (normalised to RC‐M1)	Quantity in Δ*lhaA* strain (μmol g^−1^ total protein)	Stoichiometry in Δ*lhaA* strain (normalised to RC‐M1)	Mean % change (Δ*lhaA*/WT, normalised to RC‐M1)
RC–M	RC–M1	1.20 ± 0.13	1.00	0.89 ± 0.09	1.00	0
	RC–M2	1.04 ± 0.14	0.87	0.94 ± 0.12	1.06	+22
RC‐L	RC‐L1	1.14 ± 0.20	0.95	0.92 ± 0.11	1.03	+8
	RC‐L2	1.15 ± 0.15	0.96	0.92 ± 0.09	1.03	+7
LH1 α	LH1 α	9.67 ± 1.27	8.06	0.92 ± 0.11	1.03	−87
PufX	PufX	0.92 ± 0.11	0.77	0.27 + 0.07	0.30	−61

The analyses were performed in biological triplicate with two technical replicates. Means and standard deviations are for *n* = 6. The tryptic peptides are listed in Table S2.

### Construction of the FLAG‐LhaA and FLAG‐PucC strains and their use in pull‐down assays

**Figure 5:**
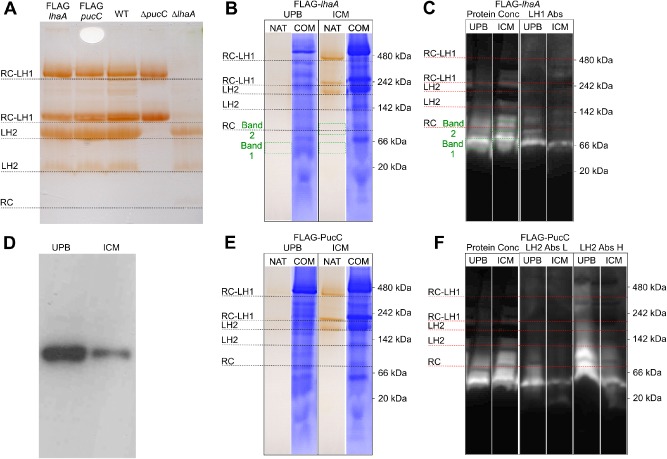
CN‐PAGE analysis of FLAG‐LhaA and FLAG‐PucC complexes in precursor (UPB) and mature (ICM) photosynthetic membranes. A. CN‐PAGE of the WT, FLAG‐LhaA and FLAG‐PucC strains, with assignments in terms of complexes indicated on the left. B. Comparison of the native and Coomassie‐stained gel profiles for UPB and ICM fractions, purified from FLAG‐LhaA cells grown under high light conditions. NAT indicates Native‐PAGE and COM indicates a Coomassie stained gel. Mass spectrometry confirmed that ICM ‘Band 1’ and ‘Band 2’ and UPB ‘Band 1’ contain LhaA (see also Table S6). C. Immunoblots of the CN‐PAGE gel lanes for the FLAG‐LhaA UPB and FLAG‐LhaA ICM samples, with loadings normalised either to total protein concentration or to the 875 nm absorption associated with the LH1 complex. D. Immunoblot of proteins separated by SDS‐PAGE of UPB and ICM membranes prepared from photosynthetically grown WT cells. The membranes were loaded for equal BChl content. The blot was probed with antibodies to LhaA. E. Comparison of the native and Coomassie‐stained gel profiles for UPB and ICM fractions, purified from FLAG‐PucC cells grown under high light conditions. UPB and ICM purified from FLAG‐PucC cells produced under high light conditions were compared by CN‐PAGE. F. Immunoblots of the CN‐PAGE gel lanes for the FLAG‐PucC UPB and FLAG‐PucC ICM samples, with loadings normalised either to total protein concentration or to the 850 nm absorption peak associated with the LH2 complex. High (H) and low (L) loadings of the 850 nm normalised membranes were used.

Having established the effects of deleting *lhaA* and *pucC* on this bacterial photosystem, strains were constructed so that pull‐down assays could be used to retrieve FLAG‐LhaA or FLAG‐PucC together with any respective near neighbours in the membrane. Primers used to create and confirm these strains are listed in Table S1. In order to maintain the normal levels of expression of the respective genes, each N‐terminal tag was constructed by insertion of the altered *lhaA* or *pucC* gene into the native position in the genome (see Fig. 1), verified by sequencing (not shown). Absorbance spectra (not shown) and clear native PAGE (Fig. 5A) were used to confirm the near wild‐type phenotype of the mutants.

Membranes from the FLAG‐LhaA strain were solubilised with a range of β‐DDM concentrations in order to find an optimum for releasing complexes from the membrane while retaining specific protein–protein interactions. The extracts were then applied to an anti‐FLAG column, followed by extensive washing with 0.04% β‐DDM. FLAG‐LhaA and any associated proteins were desorbed with FLAG peptide, digested with trypsin and the resultant peptides analysed by nano‐flow liquid chromatography coupled to a mass spectrometer. The MS and MS/MS spectra were used to identify proteins that co‐immunoprecipitated with FLAG‐LhaA (Table S3). In addition to LhaA, 38, 57, 46, 50 and 56 proteins were detected in the extracts at 0.1, 0.5, 1.0, 1.5 and 3.0% β‐DDM respectively. Out of the 95 proteins identified in total, 64 occurred at two or more β‐DDM concentrations. Therefore, there was no optimal β‐DDM concentration that produced an exclusive list of LhaA interaction partners. Proteins relevant to photosynthesis that were captured by FLAG‐LhaA were the three reaction centre subunits and the associated PufX polypeptide, LH2‐β and the cofactor biosynthesis enzymes BchE, BchI, BchP, CrtA, CrtI and HemB. Also detected were the protein translocase subunits SecE and YajC together with the membrane protein processing protease FtsH. In contrast, co‐immunoprecipitation with FLAG‐PucC (Table S4) resulted in the detection of only 21 proteins in addition to PucC itself, including LH2‐β, RC‐H and –M, BchE and BchI. LH2‐β and PucC also occurred in the anti‐FLAG negative control (Table S5), but with significantly lower database search scores than in the co‐immunoprecipitation assays, implying a low‐level non‐specific binding of these proteins by the anti‐FLAG resin. In the FLAG‐PucC assay, the RC‐H score was comparable with that in the negative control. Tables S3 and S4 show that the majority of proteins captured in these FLAG co‐immunoprecipitation assays were of diverse function with no clear direct relationship with the photosynthetic apparatus, for example ribosomal proteins and metabolic enzymes.

### Identification of PucC and LhaA complexes using clear native PAGE

Clear native PAGE (CN‐PAGE) provides a different method for investigating associations of LhaA and PucC with partner proteins, on the basis of co‐migration through a non‐denaturing gel. The membranes were pre‐solubilised with 2% β‐DDM, and the electrophoretic separation was performed at 4°C in the presence of 0.02% β‐DDM to minimize artifactual aggregation while maintaining native interactions. Figure 5A shows that this procedure separates the major photosystem complexes into RC‐LH1‐PufX dimers (∼480 kDa), monomers (∼240 kDa), a major LH2 band at ∼210 kDa and a minor one at ∼140 kDa, and finally a faint RC band at ∼80 kDa just visible in the Δ*lhaA* lane in Fig. 5A. This CN‐PAGE analysis confirms that the attachment of the N‐terminal FLAG tag does not alter photosystem stoichiometry; the Δ*pucC* and Δ*lhaA* lanes show the expected LH2‐minus and RC‐LH1‐PufX‐minus profiles.

Figure 5B and E compares the CN‐PAGE analyses of mature intracytoplasmic membranes (ICM) and the sites of initiation of membrane invagination [upper pigmented band (UPB)] that are enriched in proteins involved in pigment and protein biogenesis (Niederman *et al*., 1979; Woronowicz and Niederman, 2010; Jackson *et al*., 2012). The sample loadings were balanced for protein content, as seen in the Coomassie stained lanes, so there is far less pigment in the corresponding unstained UPB lanes, reflecting their cellular origin as indentations of the non‐photosynthetic cytoplasmic membrane (Inamine *et al*., 1984; Tucker *et al*., 2010). Figure 5B,E analyse the FLAG‐LhaA and FLAG‐PucC strain, respectively; Fig. 5C and F show immunoblots of these gels probed with anti‐FLAG antibodies, with the FLAG tag acting as a convenient marker for the positions of FLAG‐LhaA/FLAG‐PucC in the gels. UPB and ICM sample loadings in Fig. 5C and F were balanced either for protein (the two left‐hand lanes) or for LH1 absorbance (right hand lanes); equal protein loading approximates to total membrane, whereas equal LH1 absorbance approximates to the number of LH1 complexes in each sample. The outcomes are different because of the lower pigment content of UPB membranes which, as already mentioned, have their origin in respiratory cytoplasmic membranes. The CN‐PAGE immunoblot in Fig. 5C shows a larger signal for the UPB fraction, indicating more FLAG‐LhaA. The enrichment of LhaA in the UPB was confirmed by immunoblot analysis of UPB and ICM prepared from photosynthetically grown WT cells probed with an antibody to LhaA (Fig. 5D). The samples were loaded on the basis of equal BChl concentration, which is similar to the equal LH1 absorbance loadings in Fig. 5C. FLAG‐LhaA in Fig. 5C migrates mainly at ∼66 kDa, with evidence for a smaller population of oligomers as a series of bands extending to the 142–242 kDa range. In order to identify the co‐migrating proteins, Bands 1 and 2, delineated by green dotted lines, were excised from the native gel for the FLAG‐LhaA ICM sample, and Band 1 was excised from the UPB gel. Proteomic analysis by mass spectrometry on these three bands (Table S6) revealed distinct subpopulations of proteins for the UPB and ICM membranes. Of particular interest for the precursor UPB fraction are the co‐migration of FLAG‐LhaA with BChl synthase (BchG), FtsH, various ribosomal proteins and YidC, which binds to the SecYEG protein‐conducting channel in *Escherichia coli* (Sachelaru *et al*., 2013).

A similar CN‐PAGE/immunoblot analysis of FLAG‐PucC within UPB and ICM fractions (Fig. 5E and F) showed a major band at ∼50 kDa and series of higher molecular mass complexes particularly in the UPB fraction loaded with a higher concentration of material, labelled in Fig. 5E as LH2 Abs H.

### Proximity between LhaA and RC complexes probed using lifetime microscopy

**Figure 6:**
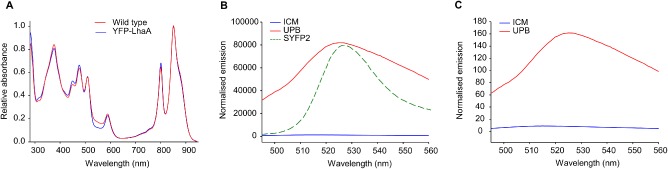
Absorption and fluorescence emission of membranes from the YFP‐LhaA strain. A. Absorbance spectra of membranes from photosynthetically grown WT (red) and YFP‐LhaA (blue) strains, normalised to 850 nm. B. Fluorescence emission spectra of UPB and ICM membrane fractions prepared from phototrophic cells grown under low light conditions; the spectra were normalised to LH1 absorbance at 875 nm. A fluorescence emission spectrum of purified SYFP2 has been overlaid (green dashed line). C. Fluorescence emission spectra of UPB and ICM membrane fractions prepared from phototrophic cells grown under low light conditions; the spectra were normalised to total protein concentration. **This figure is available in colour online at** wileyonlinelibrary.com.

Both the FLAG pulldown and CN‐PAGE analyses suggest an association between FLAG‐LhaA and RC subunits; as these are both biochemical tests, requiring disruption of membrane integrity with detergents, we used a spectroscopic assay of intact membranes to examine this association. An assay was devised based on energy transfer between the Cyan Fluorescent Protein (CFP) and the Yellow Fluorescent Protein (YFP) variant SYFP2; this pair of fluorescent proteins is suitable for assays of proximity based on Forster resonance energy transfer (FRET) (Piston and Kremers, 2007). The *syfp2* gene was fused to the 5′ end of *lhaA* and the construct integrated into the genome as already described for the FLAG‐tagged mutants; the phenotype of the YFP‐LhaA strain was unaffected, as judged by its ability to grow photosynthetically and the normal LH2/LH1 photosystem stoichiometry (Fig. 6A). As expected from the relative abundance of LhaA in the UPB sites where membrane invagination starts (Fig. 5C), YFP fluorescence was overwhelmingly found in the UPB and not in the ICM fraction, both on the basis of LH1 content (Fig. 6B) or protein content (Fig. 6C). To act as a control for the FRET measurements the *cfp* gene was fused to the 5′ end of *lhaA*, with the same outcomes as in Fig. 6A–C in terms of unaffected photosystem stoichiometry and relative abundance of CFP fluorescence in the UPB membrane fraction (results not shown).

**Figure 7:**
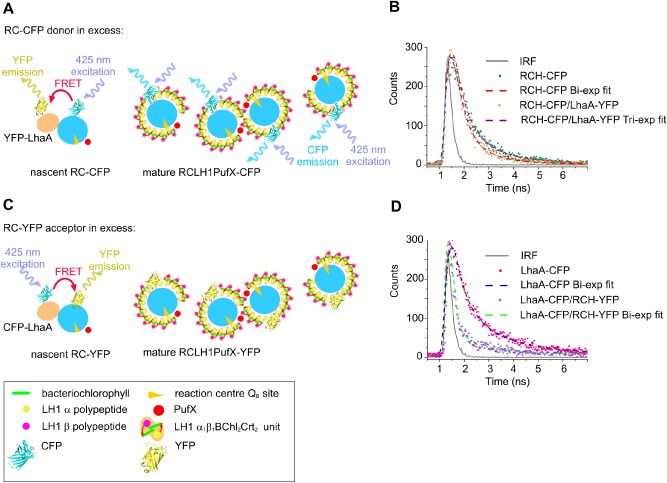
Lifetimes of CFP‐LhaA and CFP‐RC energy transfer donors. A. Diagram depicting the likely imbalance between the RC‐CFP energy transfer donors and the YFP‐LhaA acceptors in the UPB membrane. Most of the CFP fluorescence decays relatively slowly on a nanosecond timescale due to the lack of acceptors. LhaA is shown as associating with RCs at an early stage in assembly of the RC‐LH1‐PufX complex, before encirclement of LH1 subunits. B. Typical decay curves recorded at 480 ± 12 nm (CFP emission band) on control membranes containing RCH‐CFP only, membranes RCH‐CFP/LhaA‐YFP and the instrument response function (IRF) of the lifetime imaging setup, which is approximately 0.18 ns. The RC‐CFP only membranes show a double exponent decay with an amplitude averaged lifetime of 0.82 ns while the best fit for the RCH‐CFP/LhaA‐YFP membranes is tri‐exponential; the amplitude averaged lifetime is 0.62 ns. C. Diagram showing that with a limited number of CFP‐LhaA energy transfer donors most of the excitation energy is delivered to a nearby acceptor, which shortens the fluorescence lifetime of CFP‐LhaA. As in A, LhaA is shown associating with RCs at an early stage in assembly of the RC‐LH1‐PufX complex, before encirclement of LH1 subunits. D. Typical decay curves recorded at 480 ± 12 nm (CFP emission band) on control membranes containing LhaA‐CFP only, membranes with LhaA‐CFP together with RCH‐YFP, and the instrument response function (IRF). The LhaA‐CFP only membranes show a double exponent decay with an amplitude averaged lifetime of 0.89 ns, whereas the best fit for the LhaA‐CFP/RC‐YFP membranes shows a double exponent decay with an amplitude averaged lifetime of 0.26 ns.

A separate series of genomic constructs fused either the *syfp2* or *cfp* gene to the 3′ end of *puhA* encoding the RC‐H subunit, or to the 5′ end of *lhaA*. The resulting strains grew photosynthetically and RC levels were unaffected (results not shown). Finally, CFP‐LhaA/RC‐YFP and RC‐CFP/YFP‐LhaA genomic combinations were created for FRET assays to examine proximity between LhaA and RCs. Membranes were prepared from single and double tagged strains and analysed using a home‐built fluorescence lifetime microscope (see *Experimental procedures*) that records lifetimes in the 0.2–6 ns time domain, suitable for fluorophores such as CFP and YFP. FRET between CFP and YFP should shorten the fluorescence lifetime of the CFP donor; we recorded CFP lifetimes at a central wavelength of 480 nm for membranes containing the RC‐CFP/YFP‐LhaA (Fig. 7B) and CFP‐LhaA/RC‐YFP combinations (Fig. 7D), and compared them with the RC‐CFP and LhaA‐CFP controls.

Figure 7B (dark cyan data points) shows the fluorescence decay curve for membranes containing only the CFP donor coupled to the RC H‐subunit (sample RC‐CFP), recorded at a central wavelength of 480 nm. The decay is clearly bi‐exponential, as expected from previous time‐resolved studies of CFP in mammalian and fungal cells (Tramier *et al*., 2002; Becker *et al*., 2004; Duncan *et al*., 2004; Millington *et al*., 2007). The two intrinsic lifetime components likely originate from two different conformations of the CFP chromophore (Seifert *et al*., 2002; Bae *et al*., 2003), which can coexist and interchange on a millisecond timescale (i.e. much slower than the fluorescence decay timescale). The best fit for our data (Fig. 7B – red dashed line) was achieved by using double‐exponent decay function where the long‐lived component has an amplitude contribution A_1_ = 0.18 ± 0.02 and lifetime *τ*_*1*_ = 1.9 ± 0.22 ns, whereas the short‐lived component has an amplitude contribution A_2_ = 0.82 ± 0.02 and a lifetime *τ*_*2*_ = 0.58 ± 0.03 ns (*χ_red_* ^2^ = 0.91). All of the lifetimes and amplitudes are collated in Table 2. When YFP‐LhaA is present as a potential energy transfer acceptor (Fig. 7B – orange data points), RC‐CFP fluorescence exhibits an extra, fast‐decaying component with a significant amplitude contribution A_3_ = 0.41 ± 0.1 and a short lifetime *τ*_3_ = 0.32 ± 0.05 ns (Table 2). The best fit for this decay curve is therefore a triple‐exponent decay function (Fig. 7B – brown dashed line, *χ_red_* ^2^ = 0.97) where the longest‐lived component has an amplitude contribution A_1_ = 0.22 ± 0.04 and lifetime *τ*_*1*_ = 1.36 ± 0.12 ns, and the second component has A_2_ = 0.37 ± 0.1 and *τ*_2_ = 0.51 ± 0.07 ns. The presence of the third distinct, fast component in the data from the RC‐CFP/LhaA‐YFP sample, together with the similarity of this lifetime to the short lifetime component of the RC‐CFP sample, is a clear indication for the existence of two distinct RC‐CFP donor populations, depicted in Fig. 7A. Some RC‐CFP complexes are proximal to LhaA‐YFP acceptors, but the excess of RC‐CFP donors over the LhaA‐YFP acceptors creates a large proportion of unquenched CFP donors that do not contribute to the FRET. Similar results were already reported for the CFP‐YFP FRET pair (Becker *et al*., 2004; Millington *et al*., 2007).

**Table 2:** Amplitudes and lifetimes from the FRET experiments with LhaA and RC‐H labelled with either CFP or YFP

Sample	A_1_	*τ_1_* [ns]	A_2_	*τ_2_* [ns]	A_3_	*τ_3_* [ns]	*τ_av_* [ns]	*χ_red_* ^2^
RC‐CFP	0.18 ± 0.02	1.9 ± 0.22	0.82 ± 0.02	0.58 ± 0.03			0.82	0.91
LhaA‐CFP	0.32 ± 0.04	1.74 ± 0.10	0.68 ± 0.04	0.5 ± 0.06			0.89	1.05
RC‐CFP LhaA‐YFP	0.22 ± 0.04	1.36 ± 0.12	0.37 ± 0.1	0.51 ± 0.07	0.41 ± 0.10	0.32 ± 0.05	0.62	0.97
LhaA‐CFP RC‐YFP	0.07 ± 0.02	1.57 ± 0.15	0.93 ± 0.02	0.16 ± 0.04			0.26	1.02

When the ratio between the donors and the acceptors was inverted, depicted in Fig. 7C, the YFP acceptors (on the RC H‐subunit) were likely in excess of the CFP donors (on the LhaA), and the decay behaviour of the CFP significantly changed (Fig. 7D – purple data points). Now the long‐lived component, A_1_ = 0.07 ± 0.02 and *τ_1_* = 1.57 ± 0.15 ns, makes only a small contribution, and there is a new, short‐lived dominant component, A_2_ = 0.93 ± 0.02 and *τ_2_* = 0.16 ± 0.04 ns (Fig. 7D – green dashed line, *χ_red_* ^2^ = 1.02; see also Table 2). We used an LhaA‐CFP only mutant as a control sample (Fig. 7D, pink data points). The best fit for the data in this case (Fig. 7D – dark blue dashed line) was achieved by using double‐exponent decay function where the long‐lived component has an amplitude contribution A_1_ = 0.32 ± 0.04 and lifetime *τ*_*1*_ = 1.74 ± 0.1 ns, whereas the short‐lived component has an amplitude contribution A_2_ = 0.68 ± 0.04 and a lifetime *τ*_*2*_ = 0.5 ± 0.06 ns (*χ_red_* ^2^ = 1.05).

The donor‐acceptor energy transfer efficiency, *E*, can be calculated according to:

$$E=1-\frac{\tau_{DA}}{\tau_{D}}$$

where *τ_D_* is the donor lifetime and *τ_DA_* is the lifetime in the presence of the acceptor. Based on the amplitude averaged lifetimes of the LhaA‐CFP sample, *τ*_*Dav*_ = 0.89 ns, and the LhaA‐CFP/RC‐YFP sample, *τ*_*Dav*_ = 0.26 ns (Table 2), we calculate that the FRET efficiency is around 70%. This high value indicates a close association between the donor and acceptor proteins. The bulky nature of FPs can prevent a close approach between the chromophores, limiting FRET efficiencies to ∼40% (Piston and Kremers, 2007), although some studies have found values of up to 70% for closely tethered CFP‐YFP constructs (Thaler *et al*., 2005; Chen *et al*., 2006; Millington *et al*., 2007; Grünberg *et al*., 2013).

The donor–acceptor distance, *r*, can be calculated using the measured efficiency and the Förster distance, *R*_*0*_, at which energy transfer efficiency is 50%:

$$r=R_{0}\;{\left(\frac{1}{E}-1\right)}^{\frac{1}{6}}$$

**Figure 8:**
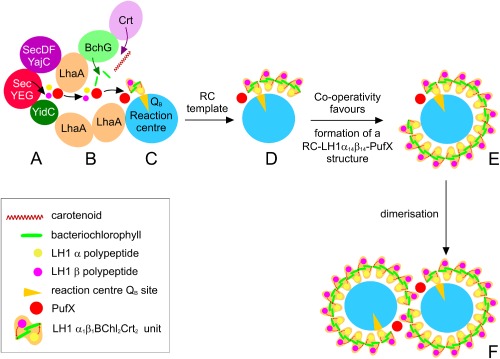
Schematic diagram depicting the proposed physical and functional linkage between components for photosystem assembly and the sequence for formation of the RC‐LH1‐PufX dimer complex within the UPB membrane. A. The SecYEG/SecDFYajC/YidC machinery for co‐translational insertion of nascent photosystem polypeptides. B. LhaA proteins link the Sec complex to the terminal enzymes of the BChl and carotenoid biosynthetic pathways, and lie close to the RC complex. The LH1 polypeptides and pigments are proposed to be sequestered within a lipid‐filled channel that fosters interactions between polypeptide and pigment components, resulting in a α_1_β_1_(Bchl)_2_Crt_2_ subunit. For simplicity, carotenoids are omitted from subsequent diagrams. The red arrows within a single α_1_β_1_(Bchl)_2_ Crt_2_ subunit indicate the hydrogen bonds between the LH1 α‐ and β‐bound BChls to αTrp43 and βTrp47, respectively (Olsen *et al*., 1994; Sturgis *et al*., 1997). C. The attachment of PufX‐LH1α_1_β_1_ to the RC initiates ring formation. The specificity of attachment of PufX to the extrinsic C‐terminal domain of the RC‐H subunit establishes the location of a pore in the LH1 ring for quinol/quinone exchange at the RC Q_B_ site. D. The RC acts as a template for further LH1α_1_β_1_ subunits that associate with one another and gradually wrap round the RC. E. A fully assembled monomeric RC‐PufX‐LH1α_14_β_14_ complex, with PufX preventing complete encirclement of the RC. F. Core monomers can dimerise, a tendency that depends partly on the carotenoids present; WT membranes with spheroidene as the major carotenoid contain mainly dimers (Chi *et al*., 2015). **This figure is available in colour online at** wileyonlinelibrary.com.

*R_0_* for the CFP‐YFP donor‐acceptor pair is approximately 5.0 nm (Tsien, 1998), and for *E* = 70%, we calculated a donor‐acceptor distance of approximately 4.1 nm. Such a small donor‐acceptor distance between CFP and YFP indicates that these proteins, both on the cytoplasmic face of the UPB membrane, are in close proximity. Thus, the carrier proteins, the LhaA and the RC H‐subunit, respectively, are closely spaced within the membrane bilayer, as depicted in the diagrams in Figs. 7 and 8.

## Discussion

### LhaA and PucC are important though not essential for LH1 and LH2 assembly, respectively

Deletion of *lhaA* significantly affects LH1 assembly, as shown by absorption spectroscopy and fractionation of detergent solubilised complexes on sucrose density gradients. Quantitative mass spectrometry (Table 1) showed that 14% of the LH1α polypeptide is retained in the Δ*lhaA* mutant, consistent with the presence of low levels of monomeric and dimeric RC‐LH1‐PufX complexes (Fig. 4). LhaA is, however, specific for assembly of the LH1 component of the RC‐LH1‐PufX complex, and RC levels are unaffected in the Δ*lhaA* mutant. The halving of PufX levels (Table 1) could be a direct effect of deleting LhaA, or more likely an indirect consequence of losing the LH1 interactions that stabilise PufX (Recchia *et al*., 1998; Qian *et al*., 2008; 2013). Similar results were obtained for the Δ*pucC* mutant, where LH2 levels were significantly affected, but low LH2 levels were detected in sucrose density gradients and immunoblots (Fig. 2).

The retention of some LH complexes in the Δ*lhaA* and Δ*pucC* mutants appears to rule out an obligatory, mechanistic role for these integral membrane proteins in LH assembly; instead, they might corral carotenoid and BChl cofactors, together with LH polypeptides within sub‐domains, to be consistent with later examples of the photosynthetic membrane, greatly enhancing the chances of these reactants combining productively. Given that bacterial photosynthetic membranes are crowded, consisting of at least 70% protein, some productive interactions between polypeptide and pigment components could be expected even in the absence of LhaA/PucC. LH1 α_1_β_1_(Bchl)_2_ units can be reconstituted *in vitro* from the separate polypeptide and BChl components (Parkes‐Loach *et al*., 1988; Davis *et al*., 1995; Todd *et al*., 1999), then assembled into α_16_β_16_(Bchl)_32_ LH1 rings (Karrasch *et al*., 1995; Harris *et al*., 2013), and carotenoids can also be reconstituted (Fiedor *et al*., 2004), supporting the idea that as long as the correct conditions are met assembly of LH1 α_1_β_1_(Bchl)_2_ carotenoid units, then oligomerisation to form LH1 complexes, will proceed. The situation with LH2 is more problematic, as *in vitro* reconstitutions succeed only rarely (Todd *et al*., 1998), possibly because the requirement for carotenoids is more exacting (Lang and Hunter, 1994). Further exploration of the necessity for *puc1BAC* and *puc2BA* operons (Zeng *et al*., 2003) and what controls the size of LH2 rings is required. Little is known about LH2 assembly other than the obligatory role of carotenoids in this process (Lang and Hunter, 1994). The LH2 antenna forms after RC‐LH1‐PufX core complexes have been assembled (Niederman *et al*., 1979; Pugh *et al*., 1998; Hunter *et al*., 2005; Koblízek *et al*., 2005).

### The initial events of core complex assembly and establishment of a RC‐PufX‐LH1α_1_β_1_ complex

Pugh *et al*. (1998) proposed that PufX had the binding order preference: RC:LH1 > LH1 > RC‐only. The immunoblot in Fig. 4D confirms that PufX binds primarily to LH1 rather than RCs, consistent with the crystal structure of the RC‐LH1‐PufX dimer showing that PufX makes close contacts with the N‐termini of the first LH1 αβ pair on the cytoplasmic side of the membrane (Qian *et al*., 2013). Early in the assembly pathway (Fig. 8B) PufX must associate with the extrinsic C‐terminal domain of the RC‐H subunit (Qian *et al*., 2013). Once docked in position, the transmembrane domain of PufX prevents any newly arriving LH1 αβ subunits from completely encircling the RC, establishing a pore that allows quinone/quinol traffic to traverse the LH1 ring and access to the RC Q_B_ site (Qian *et al*., 2013). Thus, core complex assembly is likely initiated by the formation of a compact RC‐PufX‐LH1α_1_β_1_ complex prior to encirclement by LH1 α and β polypeptides, as originally proposed (Pugh *et al*., 1998). The native gel, pulldown, mass spectrometry and FRET data suggest that LhaA, in close proximity to the RC, might recruit the LH1 α and β polypeptides and PufX in order to form an initial RC‐PufX‐LH1α_1_β_1_ complex (Fig. 8C). In its absence, monomeric and dimeric core complexes are still assembled to completion (Fig. 4C, and shown diagrammatically in Fig. 8E and F) but at a much reduced rate. By itself, PufX is not essential for initiating ring formation, as PufX‐minus mutants are still capable of producing extensive arrays of RC‐LH1 core complexes, albeit in a monomeric form (Siebert *et al*., 2004; Frese *et al*., 2008; Adams *et al*., 2011).

### A cooperative assembly mechanism ensures that once encirclement of RCs by LH1 αβ subunits is initiated, it proceeds to completion

It is surprising that the Δ*lhaA* mutant assembles normal RC‐LH1‐PufX core complexes with their full complement of encircling LH1 subunits, even though the absolute levels of RC‐LH1‐PufX core complexes are greatly reduced. Apparently, the few LH1αβ subunits that do assemble in the Δ*lhaA* mutant are not shared equally and instead, once the first LH1 αβ subunit attaches to a RC, further subunits are added until this RC is encircled, even at the expense of the other RCs present. Such a cooperative mechanism was proposed recently, on the basis of an AFM study of a mutant with LH1 levels lowered by ∼40%, a consequence of reducing levels of the cognate *pufBA* transcript (Olsen *et al*., 2014). AFM topographs of mutant membranes revealed a mixture of complete RC‐LH1 core complexes, empty LH1 rings and isolated RCs, consistent with a cooperative process for RC‐LH1αβ associations. However, in the present work, LH1 levels are much lower, yet still we observe only native RC‐LH1‐PufX complexes, along with a substantial amount of free RCs with no LH1 subunits attached.

As LH1 αβ subunits progressively wrap around RCs they necessarily form curved complexes, yet AFM studies with LH1‐only mutants of *Rba. sphaeroides* (Bahatyrova *et al*., 2004b; Olsen *et al*., 2014) and pigment reconstitutions with LH1 α and β polypeptides (Karrasch *et al*., 1995; Harris *et al*., 2013) both show that LH1 subunits are inherently able to form ring structures in the absence of RCs. The recent AFM study of membranes prepared from an LH1‐only mutant showed that LH1‐only complexes largely consist of closed circular structures of 15–16 α_1_β_1_(Bchl)_2_ subunits; however, ellipses, arcs and spirals were also observed, showing that the RC acts as a template for sizing and shaping the LH1 ring. In this sense, the RC can be regarded as an assembly factor for the LH1 complex. Nevertheless, in strains capable of normal RC‐LH1‐PufX assembly some ‘empty’ LH1 rings with no enclosed RCs have been observed both spectroscopically and in AFM topographs (Olsen *et al*., 2014). We have proposed that the RC‐LH1‐PufX assembly pathway is biased towards an excess of LH1 polypeptides over RCs, manifested as empty LH1 rings (Olsen *et al*., 2014); consistent with this idea, quantitative mass spectrometry of membrane biogenesis centres from *Rba. sphaeroides* showed a level of the LH1 α polypeptide twofold higher than that required to form a RC‐LH1‐PufX complex (Olsen *et al*., 2014). Surplus LH polypeptides during RC‐LH1‐PufX assembly avert the deleterious consequences of unattached BChls, and the occasional assembly of LH1‐only rings carries no penalty; such complexes can still harvest energy and pass it on to RC‐LH1‐PufX complexes (De Rivoyre *et al*., 2010).

### Developing nascent photosynthetic membranes are enriched in PucC and LhaA, each of which forms oligomers

Analysis of UPB membranes from the FLAG‐LhaA and FLAG‐PucC strains by clear‐native PAGE, and subsequent immunoblotting with anti‐FLAG antibodies reveal a significant enrichment of LhaA and PucC in the UPB membrane (Fig. 5C and D); this conclusion can be drawn when the samples are compared on the basis of LH1/LH2 absorption, which is reasonable given that the aim is to understand the stoichiometry of LhaA/PucC in relation to their LH1 or LH2 products. Normalising to total protein lowers the relative level of LhaA and PucC, as it takes into account the much greater number of non‐photosynthetic proteins in the UPB fraction (Jackson *et al*., 2012). The enrichment of LhaA in the UPB membrane is more obvious in the YFP‐LhaA strain examined in Fig. 6, but neither the immunoblot nor the YFP labelling approaches are quantitative in these experiments, and comparison between these two detection methods is not possible. Qualitatively, it is clear that LhaA/PucC are found preferentially in UPB membranes, consistent with the role of the UPB as a site of initiation of membrane invagination originally proposed on the basis of pulse‐chase radiolabelling (Niederman *et al*., 1979) and consistent with a series of spectroscopic, morphological and proteomic studies (Hunter *et al*., 1979a, 1979b; Tucker *et al*., 2010; Woronowicz and Niederman, 2010). The immunoblotting also provides the first indications of the oligomeric state of the LhaA and PucC assembly factors in membranes; the most prominent band at 50–60 kDa is likely to contain LhaA or PucC monomers, given their respective calculated molecular masses of 50327 and 48973 Da, but there is also a ladder of bands up to ∼300 kDa arising from LhaA/PucC oligomers and likely also from associations with other proteins.

### LhaA co‐isolates with pigment biosynthesis enzymes and with membrane protein translocase components

Pulldown assays with tagged LhaA or PucC as bait was used to identify possible interaction partners. FLAG‐LhaA (Table S3) captured the reaction centre H, M and L subunits and the associated core complex polypeptide PufX. Other photosynthesis‐related proteins detected were LH2‐β, the BChl biosynthesis enzymes delta‐aminolevulinic acid dehydratase (HemB), Mg‐protoporphyrin IX monomethyl ester cyclase (BchE), Mg‐chelatase 38 kDa subunit (BchI) and geranylgeranyl reductase (BchP), and the carotenoid biosynthesis enzymes phytoene desaturase (CrtI) and spheroidene monooxygenase (CrtA). Of potential additional significance was the identification of the membrane protein translocase subunits SecE and YajC, and the integral membrane protease FtsH. However, the capture of a further 81 proteins suggests that this co‐immunoprecipitation assay reveals a complex, membrane‐associated network of proteins that co‐isolates with FLAG‐LhaA with interactions strong enough to survive β‐DDM solubilisation. Therefore, rather than identifying a simple set of interacting proteins, as demonstrated by other studies (Hollingshead *et al*., 2012; Chidgey *et al*., 2014), these results suggest that LhaA is a component of a photosynthetic membrane sub‐domain. In contrast to the FLAG‐LhaA experiment pulldowns with FLAG‐PucC unexpectedly retrieved only 21 proteins (Table S4), confirming that these assays were not acting non‐specifically, otherwise we might have expected a similar number of proteins to be identified in each case. This result might reflect the occurrence of LhaA and PucC in different sub‐domains of the membrane with markedly different proteomic complexities. Another approach used to search for interactions involving LhaA was co‐migration in CN‐PAGE. Proteomic analysis of FLAG‐LhaA‐containing bands in isolated ICM and UPB fractions (Fig. 5B and C, and Table S6) showed a complement of proteins similar to those found by FLAG‐LhaA pulldowns. Potentially significant is the presence in ICM of the integral membrane protease FtsH, the protein translocase subunits YajC and TatA, together with SecD and YidC, which are associated with the SecYEG protein–conducting channel in *E. coli* (Sachelaru *et al*., 2013). These proteins were also detected in UPB, except that the Sec translocase was represented by SecE and SecG instead of SecD. As these proteins cover a wide molecular mass range, from 7.1–68.6 kDa, their co‐migration in CN‐PAGE would be unlikely unless they were components of a single complex. A recent study of ICM biogenesis in *Rba. sphaeroides* using the protonophore CCCP found a preferential accumulation of SecY, A, D, F, DnaJ, K, in the UPB membrane, consistent with our results (Woronowicz *et al*., 2015).

In a recent analysis of cyanobacterial photosystem assembly, pulldown experiments with FLAG‐tagged chlorophyll synthase (ChlG) retrieved a complex consisting of ChlG, the high light‐inducible protein HliD, the Ycf39 protein and the YidC/Alb3 insertase, a result that connected the processes of chlorophyll biosynthesis and the Sec/YidC‐dependent co‐translational insertion of nascent photosystem polypeptides into membranes (Chidgey *et al*., 2014). Although the present study cannot reveal all the direct interaction partners of LhaA, the occurrence of this LH1 assembly factor in a membrane sub‐domain alongside BchG and the membrane protein insertion apparatus suggests the possibility of a similar linkage in *Rba. sphaeroides*, depicted in Fig. 8A and B. BchG is the membrane protein that catalyses the penultimate step of BChl biosynthesis, the attachment of the geranylgeranyl tail to the bacteriochlorin macrocycle (Oster *et al*., 1997; Addlesee *et al*., 2000). The membrane protein ‘insertase’ YidC is a member of the YidC/Oxa1/Alb3 protein family involved in biogenesis of membrane proteins in bacteria, mitochondria and chloroplasts (Spence *et al*., 2004; Göhre *et al*., 2006). In *E. coli* inner membranes YajC associates with SecDF, and SecYEG‐SecDF‐YajC‐YidC form a large holotranslocon complex, which can now be purified in its active state (Schulze *et al*., 2014). Here, we propose that LhaA‐BchG‐YidC‐YajC interactions are found preferentially in the UPB membrane fraction: given that this membrane has been shown to be enriched in LhaA (Fig. 5), that it is a preferential site for biosynthesis of pigments and photosystem apoproteins (Niederman *et al*., 1976; Hunter *et al*., 2005; Tucker *et al*., 2010) and that transmission electron microscopy, tomography and atomic force microscopy show that it consists of membrane discs approximately 50 nm in diameter (Tucker *et al*., 2010), it is reasonable to suggest that photosystem assembly in *Rba. sphaeroides* is localized to these specialized, confined membrane regions that form indented regions of the cytoplasmic membrane and persist as the ICM structure develops. Thus, the present work can be aligned with our cyanobacterial study, in showing a possible connection between BChl biosynthesis, co‐translational insertion of nascent photosystem polypeptides and their folding and assembly to form photosynthetic complexes (Fig. 8). Proximity of the (B)Chl synthases to the apparatus for membrane protein insertion and photosystem assembly (Fig. 8B) might channel and protect potentially phototoxic (B)Chlide substrates and (B)Chl products and co‐ordinate the arrival of pigments and nascent apoproteins to produce photosynthetic complexes. In the case of *Rba. sphaeroides*, the UPB membrane fraction, which originates from membrane biogenesis centres, is proposed to house photosystem assembly domains where LhaA and PucC, both integral membrane proteins, assist in the assembly of LH1 and LH2 complexes respectively. Although we do not know how LhaA and PucC work, they could promote the association of nascent LH polypeptides with their carotenoid and BChl cofactors, possibly by forming oligomers that sequester the pigment and polypeptide reactants into lipid‐filled channels, represented by the diagram in Fig. 8. Such membrane nanodomains could maximise interactions between pigments newly arriving from BchG and the SecYEG‐SecDF‐YajC‐YidC assembly machinery (Fig. 8A), thereby co‐ordinating delivery of pigments and the co‐translational insertion of LH polypeptides.

## Experimental procedures

### Standard buffers, reagents and media

All buffers and culture media were prepared as described in Sambrook *et al*. (1989), unless otherwise stated. All media and solutions were prepared using distilled water purified through a Milli‐Q system (Millipore). Growth media and solutions used for DNA work were sterilized by autoclaving at 15 psi for a minimum of 20 min or by filtration through 0.2 μm filters. Heat‐labile solutions such as antibiotics and vitamins were only added to the culture medium once it had cooled to below 50°C.

### *Escherichia coli* strains and plasmids

Two strains of *E. coli* were primarily used in this work. JM109 chemically competent cells purchased from Sigma and S17‐1 (Simon *et al*., 1983). S17‐1 cells were used for plasmid transfer into *Rba. sphaeroides* strains and were made electrocompetent. Strains were grown in Luria–Bertani (LB) medium (Sambrook *et al*., 1989) with antibiotics added when required. The following antibiotic concentrations (μg ml^−1^) were used: kanamycin 30; ampicillin 200; tetracycline, 10. When grown in liquid cells were agitated at 300 rpm.

### *Rhodobacter sphaeroides* strains

Unless otherwise stated, *Rba. sphaeroides* refers to wild‐type *Rhodobacter sphaeroides* strain 2.4.1. Wild‐type and mutant strains were grown in M22+ medium (Hunter and Turner, 1988); 0.1% casamino acids was used to supplement liquid cultures. Antibiotics were used at the following concentrations (μg ml^−1^): kanamycin, 30. Stocks were stored in LB medium containing 50% glycerol (v/v) at −80°C.

### Photosynthetic growth

Anaerobic cultures of *Rba. sphaeroides* grown under photosynthetic conditions were exposed to 15 W or 20 W MEGAMAN® CFL bulbs to achieve the desired light intensity. Light intensity was measured in μmol photons s^−1^ m^2^ using a LI‐250A light meter equipped with a LI‐190 Quantum sensor (LI‐COR Biosciences). One millilitre of semi‐aerobic culture was used to inoculate a full 30 ml universal of M22+ medium. A small magnetic stir bar was placed in the bottom of the bottle, and the culture was incubated in the desired light intensity, overnight with gentle agitation. This culture was used to inoculate either a 500 ml medical flat or a 1.2 l Roux culture bottle filled with M22+ medium and capped with a rubber bung. These cultures also contained a magnetic stir bar to allow for gentle agitation.

### Construction of genomic deletion mutants

This procedure is described in Chi *et al*. (2015). Briefly, pRK and pK18mob*sacB* constructs (Schäfer *et al*., 1994) were transferred by conjugation from *E. coli* strain S17‐1 to the appropriate *Rba. sphaeroides* strain. Following formation of kanamycin‐resistant transconjugant colonies, cells were grown in M22+ medium containing kanamycin then serially diluted onto a series of M22+ agar plates containing 10% sucrose. Cells that grew on the sucrose containing medium were replica plated onto M22+ with kanamycin and M22+ with no antibiotic. In theory, these cells had undergone a second homologous recombination event to excise the plasmid. Colonies that grew on the plate containing no antibiotics but failed to grow on the plate containing kanamycin were analysed by colony PCR to detect the deletion of *pucC* or *lhaA* using primers (Table S1) designed to specifically amplify the desired genomic fragment. Mutants were streaked on plates and grown for further analysis and storage.

### Cell harvesting and breakage

Cells were centrifuged at 4,000 × *g* at 4°C for 25–35 min until pelleted. Cell pellets were resuspended in various buffers depending on downstream analysis. Cells harvested for absorption spectroscopy, fluorescence spectroscopy or fractionation of the LH2, core complex monomer and dimer bands were resuspended in 20 mM HEPES, 5 mM EDTA, pH 7.5. Cells harvested for continuous gradients designed to separate mature ICMs from developing membranes (UPB) were resuspended in 1 mM Tris‐HCl, 1 mM EDTA, pH 7.5. Approximately 5 g of cells were used per 10 ml of buffer. Cells were disrupted by passage through a French pressure cell at 18 000 psi. Cells were passed twice through the pressure cell before unbroken cells were removed by centrifugation at 33 000 × *g* at 4°C for 25 min. The supernatant was transferred to a clean tube prior to loading onto a sucrose gradient.

### Standard preparation of ‘mixed’ ICMs

High concentrations of ICMs were achieved using a 15/40% (w/w) discontinuous sucrose gradient. One to five millilitres of broken cells was layered on top of the 15% sucrose band using either a peristaltic pump or pipette. Gradients were centrifuged at 27 000 rpm (60 000–65 000 × *g*) in a Beckman Type 50.2 Ti or Type 45 Ti rotor at 4°C for 10 h. A pigmented band of ICM formed at the 15/40% interface and was collected using a fixed needle and a peristaltic pump.

### Fractionation of LH2, core complex monomers and dimers present in ICM membranes

Membranes harvested from discontinuous sucrose gradients were diluted in 20 mM HEPES, pH 7.5 and pelleted at 45 000 rpm (180 000 × *g*) for 2.5 h using a Beckman Type 50.2 Ti rotor at 4°C. Pelleted cells were resuspended in approximately 100–200 μl of 20 mM HEPES, pH 7.5 and the absorbance spectrum used to record the OD_875_ value. The 7.5 OD_875_ units of resuspended membranes were solubilised in 3% β‐DDM in a total volume of 250 μl before 1 h of centrifugation at 15 000 rpm at 4°C in a refrigerated microcentrifuge. The supernatant was collected and layered on top of a discontinuous sucrose gradient containing 20%, 21.25%, 22.5%, 23.75% and 25% sucrose 20 mM HEPES and 0.03% β‐DDM. Gradients were centrifuged in a Beckman SW41 Ti rotor at 27 000 rpm (90 000 × *g*) for 40 h. Digital photos of the gradients were taken and pigmented bands were harvested for downstream processing.

### Clear native polyacrylamide gel electrophoresis (CN‐PAGE)

Protein complexes were generally separated by CN‐PAGE using precast NativePAGE™ Novex® 4–16% Bis‐Tris gels containing 10 1.0 mm wells. The anode buffer used was 25 mM imidazole pH 7.0, and the cathode buffer was 0.02% β‐DDM, 0.005% deoxycholic acid, 50 mM Tricine and 7.5 mM imidazole pH 7.0. Three hundred micrograms of sample in 112.5 μl of buffer was solubilised for 1 h on ice in the dark with 2% β‐DDM prior to centrifugation at 15 000 rpm for 30 min. The insoluble pellet was discarded, and the sample made up to contain 20% glycerol with a 50% glycerol stock. Twenty‐five microlitres of sample was added to each lane (15 μl sample, 10 μl 50% glycerol). The gel was run at a constant current of 10 mA for 6 h at 4°C.

### Western blot analysis of proteins: immunodetection

Following transfer, the nitrocellulose membrane was blocked for 30 min in blocking buffer (5% Marvel Milk Powder; 18 mM Tris‐HCl, pH 7.6; 68 mM NaCl), before incubation with the primary antibody in wash buffer overnight at 4°C. The following day membranes were washed twice in 30 ml wash buffer for 5 min at room temperature. Twenty‐five millilitres of wash buffer and 2.5 μl of secondary antibody were then added and the membrane incubated at room temperature for 1 h. The membrane was further washed 5 × 5 min in 30 ml of wash buffer before immunodetection was performed using Amersham^TM^ ECL^TM^ Western blotting analysis system (GE Healthcare Life Sciences) according to the manufacturer's instructions. Primary antibodies were used at a 1:5 000 dilution, and secondary antibodies were used at a 1:10 000 dilution unless otherwise stated.

### Gel filtration by HPLC

Analytical gel filtration by HPLC was performed using a BioSeph‐Sec‐4000 column (Phenomenex) on an Agilent 1200 HPLC. Gel filtration buffer was 20 mM HEPES pH 7.5. Elution of LH2, LH1, core complexes and reaction centres was detected by monitoring the absorbance at 800 nm.

### Immunoprecipitation of FLAG‐tagged proteins

Membranes from 2 to 4 l of photosynthetically grown cells were harvested from discontinuous sucrose gradients, diluted in 20 mM HEPES, pH 7.5 and pelleted at 45 000 rpm (180000 × *g*) for 2.5 h using a Beckman Type 50.2 Ti rotor at 4°C. Membranes were resuspended to an OD_875_ of 30 and incubated in 0.1–3.0% β‐DDM for 30 min at 4°C. Insoluble material was removed by centrifugation at 20 000 rpm (32 000 × *g*) in a Beckman JA‐25.50 rotor at 4°C. The supernatant was loaded onto a pre‐equilibrated 150 μl anti‐FLAG M2 affinity resin column. The column was washed with 15 volumes of wash buffer containing 0.04% β‐DDM. FLAG‐tagged and associated proteins were desorbed by the addition to the plugged column of 100 μg FLAG peptide dissolved in 500 μl wash buffer. The resin was transferred to a cryovial and rotated for 1 h at room temperature prior to a 1500 × *g* centrifugation for 5 min in a Costar Spin‐X centrifuge tube containing a cellulose acetate membrane with 0.22 μm pores to separate proteins from the resin.

### Preparation of FLAG eluates for analysis by mass spectrometry

After elution from the anti‐FLAG resin, proteins were reduced, S‐carbamidomethylated and digested with trypsin, followed by desalting of the resultant peptides as previously described (Hollingshead *et al*., 2012).

### Preparation of proteins resolved by CN‐PAGE for analysis by mass spectrometry

Protein bands from CN‐PAGE gels were excised and subjected to in‐gel tryptic digestion according to Pandey *et al*. (2000).

### Preparation of ^15^ N‐labelled internal standard for mass spectrometry‐based quantification

The ^15^N‐labelled internal standard was constructed as an artificial protein sequence (Pratt *et al*., 2006) comprising proteotypic tryptic peptides belonging to the target proteins (Table S2). This protein was expressed in *E. coli* and purified as described previously (Qian *et al*., 2013).

### Relative quantification of RC, LH1 and PufX proteins

Pelleted membranes were resuspended in 50 mM ammonium bicarbonate (ABC, BioUltra grade, Fluka). Assays were performed on three biological replicates containing 6 μl ^15^N‐labelled artificial standard protein (5 pmol μl^−1^ in 50 mM ABC), 1 μl 1% (w/v) ProteaseMAX surfactant (Promega), 2 μl purified membranes (containing 10 μg of protein by Bradford assay) and 1 μl 100 mM dithiothreitol. Proteins were solubilised by incubation at 56°C for 20 min. After brief centrifugation 8 μl 50 mM ABC was added followed by 2 μl trypsin (porcine, dimethylated, proteomics grade, Sigma, 0.2 g L^−1^ in 0.05% (w/v) ProteaseMax surfactant in 50 mM ABC) and the samples incubated at 48°C. After 3 h, 1 μl trypsin (freshly prepared as above) was added and the digestion allowed to proceed for a further 3 h. To hydrolyse the surfactant, 2.2 μl 5% (v/v) trifluoroactetic acid (TFA) was added, followed by 5 min incubation at room temperature. The samples were desalted on C_18_ SpinTip columns (Proteabio) according to the manufacturer's instructions and dried by vacuum centrifugation. They were stored at −20°C before analysis by mass spectrometry.

### Mass spectrometry and data processing

For quantitative analysis, samples were re‐dissolved in 10 μl 0.1% (v/v) TFA, 3% (v/v) acetonitrile. Duplicate 4 μl aliquots were injected onto an Ultimate 3000 RSLCnano liquid chromatography system (Dionex, Camberley, UK) with 5 mm × 300 μm trapping and 75 μm × 15 cm analytical PepMap C_18_ reverse phase columns. Elution of tryptic peptides was by a 90 min linear gradient from 94% solvent A (0.1% (v/v) formic acid) to 40% solvent B (0.1% (v/v) formic acid in 80% (v/v) acetonitrile) at a flow rate of 300 nl min^−1^. Mass spectra were acquired online using a Maxis UHR‐TOF instrument (Bruker Daltonics, Bremen, Germany) operating in profile mode with automated dependent MS/MS scans. Qualitative protein identification from in‐gel digests was performed using an Amazon ion trap instrument (Bruker), also with data dependent acquisition. For both types of analysis, spectra were first converted to Mascot Generic Files using scripts provided by Bruker before submission for database searching via Mascot Daemon v.2.5.1 running with Mascot Server v.2.5 (Matrix Science, London, UK) against the *Rba. sphaeroides 2.4.1.* complete proteome database (http://www.uniprot.org/proteomes/UP000002703). For protein quantification, the identities and chromatographic retention times of their expected proteotypic peptides were confirmed by the database search results (see above). Their ion intensities were then used in comparison with those of the ^15^N‐labelled internal standard peptides to calculate picomolar amounts.

### Absorption and fluorescence spectroscopy

Room temperature absorbance spectra were recorded on a Cary 50 UV‐Vis spectrophotometer. Whole cell spectra were recorded between 600 and 950 nm and spectra of membrane preparations or purified proteins were taken between 280 and 950 nm; baseline correction was performed for both. Samples were diluted as appropriate so that the spectrophotometer readings were in the range 0.1–0.8.

UV‐Vis fluorescence spectroscopy was performed with a SPEX Fluorolog spectrofluorometer (SPEX Industries) with a xenon light source. Fluorescence emission spectra recorded with excitation at 475 nm (5 nm slit width), and detection with an integration time of 1 s using a 5 nm slit width. All emission and excitation spectra were recorded in 20 mM HEPES buffer, pH 7.5.

Fluorescence emission spectra were recorded with 5 nm excitation and 2.5 nm emission slits. Excitation was performed at 475 nm. An integration time of 1 s was used, and each spectrum is an average of three scans.

### Fluorescence lifetime measurements

Fluorescence lifetime measurements were performed on a home‐built lifetime imaging microscope equipped with a spectrometer (Acton SP2558, Princeton Instruments), EMCCD camera (ProEM 512, Princeton Instruments) and a single photon hybrid photodetector (HPM‐100‐50 Becker & Hickl). A supercontinuum white light laser (SC 480‐10, Fianium) with a repetition rate of 40 MHz was used as an excitation source. The excitation wavelength was selected by a 438/24 nm band‐pass filter (FF02‐438/24‐25, Semrock) and additionally cleaned by two short‐pass filters (M254C45, Thorlabs). Fluorescence emission detection was filtered through a 458 nm dichroic mirror (FF458‐Di02, Semrock) and a 483/32 nm band‐pass emission filter (FF01‐483/32‐25, Semrock), thus selecting the light emitted only from the CFP donor labels. A secondary slit in front of the single photon detector allowed further spectral narrowing of the measured signal; typically we were able to select ± 6 nm around the central wavelength of 480 nm selected by the monochromator. The laser beam was focused on the sample surface to a diffraction limited spot using 100× objective (PlaneFluorite, NA = 1.4, oil immersion, Olympus), and the modulation of the laser was synchronised with a time‐correlated single‐photon counting module (SPC‐150, Becker & Hickl). Fluorescence lifetimes were recorded by parking the focused laser spot over a selected part of the sample surface and collecting data for 0.2 s; multiple measurements were performed on 3–15 different locations on each sample. SPCM software (Becker & Hickl) was used for the data acquisition. The families of decay curves were analysed with OriginPro and TRI2 software packages by fitting a multiexponential decay function:

$$I\left(t\right)=\sum_{i=1}^{n}A_{i}exp\left(\frac{-t}{\tau_{i}}\right)+B$$

where *τ*_i_ is the fluorescence lifetime, *A_i_* is the fractional amplitude contribution of the *i* ^th^ decay component and *B* is the background. The quality of fit was judged on the basis of the reduced *χ* ^2^ statistic:

$$\chi^{2}{}_{red}=\frac{\sum_{k=1}^{n}\frac{{\left[I\left(t_{k}\right)-I_{c}\;\left(t_{k}\right)\right]}^{2}}{I\left(t_{k}\right)}}{n-p}=\frac{\chi^{2}}{n-p}$$

where *I(t_k_*) is the data at time point *k*, *I_c_(t_k_)* is the fit at time point *k*, *n* is the number of data points and *p* is the number of variable fit parameters (*n* − *p* = degrees of freedom).

The instrument response (IRF) of the system, measured using a mirror, was approximately 0.18 ns, and the convolution of the decay curves with the IRF was taken into account when the fitting was performed.

The samples were pelleted cell membranes resuspended in imaging buffer (20 mM HEPES, pH 8) sparged with nitrogen to avoid photo‐oxidation of the CFP and YFP labels and the photosynthetic proteins. Then, the membrane suspension was deposited onto a clean glass substrate (coverslip), mounted and sealed onto a standard microscope slide.

## Supporting information

Supporting information
