# Supplementary material for: PucC and LhaA direct efficient assembly of the light‐harvesting complexes in Rhodobacter sphaeroides
Source: Mol Microbiol. 2015 Nov 5;99(2):307–27. doi: 10.1111/mmi.13235 (PMC4949548; doi:10.1111/mmi.13235)
Supplement: Supplementary file 1 — Supporting information [file MMI-99-307-s001.pdf]

Table S1. List of primers.

Name	Sequence	Cleavage site
Δ*lhaA* Up For	CCGGAATTCAGCATCAGGACCGCGTGACCT	*Eco*RI
Δ*lhaA* Up Rev	GCGCTCTAGAGATCACGGCCGGAACTCCAGA	*Xba*I
Δ*lhaA* Down For	GCGCTCTAGACCCACCTGACACCGGAGG	*Xba*I
Δ*lhaA* Down Rev	CCCCAAGCTTAGGCCGGGCCGACGCCGT	*Hin*dIII
FLAG-*lhaA* Up For	GCGCTCTAGAAGCATCAGGACCGCGTGACCTTCGCT	*XbaI*
FLAG-*lhaA* Up Rev	GCGCTCTAGACATATGGATCACGGCCGGAACTCCAGACACTC	*Nde*I
FLAG-*lhaA* Down For	GCGCTCTAGAGCGGCCGCAATGATCCTGGGGCGGAAACAGCTGAAGAAAC	*Not*I
FLAG-*lhaA* Down Rev	GCGCGTCGACGCGCGAAGGGCATGATGGCCAGC	*Sal*I
lhaA confirmation For	ATCCGGCAGACGGTGCGCGAGG	-
lhaA confirmation Rev	TTGCCCGCGATCTCGAGATCGCAGC	-
FLAG-*lhaA* Conf Rev	GATCTGGTCGCCCGACAGCACGATC	-
FLAG-*lhaA* Conf For	TCTTCGCCGGTGCGGGCCTGATC	
Δ*pucC* Up For	CCGGAATTCTCATCGCGCACTTCCTCG	*Eco*RI
Δ*pucC* Up Rev	GCGCTCTAGAGCTCATGCGTGAAGAATC	*Xba*I
Δ*pucC* Down For	GCGCTCTAGACGCCTGTGACGAATCACC	*Xba*I
Δ*pucC* Down Rev	CCCCAAGCTTAAAAGAGTCGGTCGAATG	*Hin*dIII
FLAG-*pucC* Up For	GCGCTCTAGATCATCGCGCACTTCCTCGCCGCCG	XbaI
FLAG-*pucC* Up Rev	GCGCTCTAGACATATGGCTCACGCGTGAAGAATCCCGGGGTAG	*Nde*I
FLAG-*pucC* Down For	GCGCTCTAGAGCGGCCGCAATGAGCCGAATTGCCGAACATCTGGTCCG	*Not*I
FLAG-*pucC* Down Rev	GCGCGTCGACCGGCCGAGGAGACGCCGAGC	*Sal*I
pucC confirmation For	TCTGAACAAAGTCTGGCCGAGCGGCC	-
pucC confirmation Rev	AGGGCACGACGTTCGGAGAGCCGG	-
FLAG-PucC Conf Rev	CCGCCCACCATCAGGAAGGCGAG	-
FLAG-PucC Conf For	TGGGTGCTCGGCCGGGGCTTC	-

Table S2. Proteotypic tryptic peptide sequences used to assemble the artificial ^{15}N-labelled internal standard protein.

Sequence element	Tryptic peptide	Notes
pET-14b N-terminal extension	MGSSHHHHHHSSGLVPRGSH	His-tag for purification by immobilised Ni^{2+} ion affinity chromatography
2xW	AWSWK	Additional Trp-containing sequence to increase A_{280} for protein concentration measurement
**RC–M1**	**AEYQNIFSQVQVR**	2^+ and 3^+ ion intensities added together for quantification
RC-M	FGGER	N-side flanking peptide to ELE-ADR to mimic the cleavage site in the natural protein
**RC- M2**	**ELEQIADR**	
**RC-L1**	**ALLSFER**	
**RC-L2**	**TPDHEDTFFR**	
RC-L	DLVGYSIGTLGIHR	C-side flanking peptide to TPD-FFR to mimic the cleavage site in the natural protein
**LH1 α**	**IWMIFDPR**	Met and Met sulphoxide 2^+ ion intensities added together for quantification
PufX	ADK	N-side flanking peptide to TIF-NPK to mimic the cleavage site in the natural protein
**PufX**	**TIFNDHLNTNPK**	2^+ and 3^+ ion intensities added together for quantification

The sequence elements were incorporated into the ^{15}N-labelled artificial internal standard protein in the order specified in this Table. Proteotypic peptides that were used in the quantification of the target proteins are highlighted in bold. For the reaction centre subunits M and L, 2 peptides were selected on the basis of their frequent identification in proteomic analyses of *Rba. sphaeroides* intracytoplasmic membranes. Although selection of ≥2 peptides per protein is advisable, only 1 peptide is proteotypic for LH1 α and PufX because of the numbers and locations of tryptic cleavage sites in these polypeptides. Some sites digest only poorly with trypsin, but prolonged treatment gives 99.4% digestion after 6 hours for both natural and artificial proteins.

Table S3. Proteins identified from FLAG-LhaA co-immunoprecipitation assays.

Gene name	Protein name	MOWSE score/Significant peptide count at % β-DDM				
		0.1	0.5	1.0	1.5	3.0
atpA	ATP synthase subunit alpha*	399/9	316/8	286/7	579/9	150/5
atpD1	ATP synthase subunit beta 1	163/7	281/7	114/3	94/3	265/8
atpF2	ATP synthase subunit b2		102/2			
bamA	Outer membrane protein assembly factor BamA	77/1	142/5	149/3	110/4	209/6
bchE	Anaerobic Mg-protoporphyrin IX monomethyl ester cyclase		50/3	68/3	119/4	86/3
bchI	Magnesium-chelatase 38 kDa subunit		62/2	60/3	73/2	23/2
bchP	Geranylgeranyl hydrogenase		115/3	65/2		41/2
cbbL	Ribulose bisphosphate carboxylase large chain	78/1				
cbbM	Ribulose bisphosphate carboxylase	46/2				70/1
cbbX	CbbX protein		55/1	61/3	129/3	46/2
cycA	Cytochrome c_2	97/1	65/1			
~~cobs~~	Cobalamin synthase					74/2
crtA	Spheroidene monooxygenase		80/1	50/1		
crtI	Phytoene desaturase	54/1	79/2		75/4	132/3
comL	Outer membrane protein assembly factor BamD*		87/2			
eno	Enolase	160/3	112/3			
exoP	Putative succinoglycan biosynthesis transport protein*		198/5	90/4	327/7	176/3
fbcC	Cytochrome c_1				52/1	
fliO	Flagellar protein FliO				75/2	
ftsH	ATP-dependent zinc metalloprotease FtsH		52/1	104/3	84/2	115/4
glnA1	Glutamine synthetase*	55/1			52/1	128/2
groL1	60 kDa chaperonin 1	501/10	566/14	195/6	365/7	49/4
hemB	Delta-aminolevulinic acid dehydratase					56/2
metK	S-adenosylmethionine synthase				47/2	77/3
nuoC	NADH-quinone oxidoreductase subunit C/D					38/2
nuoE1	NADH dehydrogenase subunit				22/2	
nuoF1	NADH dehydrogenase 51 kDa subunit					85/2
nuoG1	NADH dehydrogenase gamma subunit				72/1	342/6
nuoN	NADH-quinone oxidoreductase subunit N			50/1		
panB	3-methyl-2-oxobutanoate hydroxymethyltransferase	60/2	54/1		54/1	
prkB	Phosphoribulokinase	68/1				
PSrp1	Ribosomal subunit interface protein Y	79/1	64/1		50/2	100/1
pucB	Light-harvesting protein B-800/850 beta chain*	156/2	209/2	224/2	162/2	162/2
pufL	Reaction center protein L chain	67/2	70/3	82/3		48/2
pufM	Reaction center protein M chain		158/2	179/2	99/2	42/2
pufX	Intrinsic membrane protein PufX		58/1	108/1		
puhA	Reaction center protein H chain*	63/3	123/3	100/4	46/2	32/2
rhlE	ATP-dependent helicase, DEAD-box			42/2	118/3	
Rho	Transcription termination factor Rho				51/1	
rplB	50S ribosomal protein L2	147/3	99/2	61/2		120/2
rplF	50S ribosomal protein L6	52/2	61/2			

rplI	50S ribosomal protein L9	95/1	110/1			
rplL	50S ribosomal protein L7/L12		62/1	94/1		
rplP	50S ribosomal protein L16	92/1	127/1	80/1		65/1
rplQ	50S ribosomal protein L17					54/1
rplS	50S ribosomal protein L19	65/1		58/1		
rplU	50S ribosomal protein L21	36/2				
rplY	50S ribosomal protein L25	72/3	41/2	28/2		
rpsB	30S ribosomal protein S2	38/2	58/2	43/2	125/5	70/2
rpsC	30S ribosomal protein S3		155/5	57/3		171/5
rpsD	30S ribosomal protein S4*	90/3	73/2	36/2		72/3
rpsG	30S ribosomal protein S7*		52/2			
rpsI	30S ribosomal protein S9*				44/2	
rpsK	30S ribosomal protein S11		98/1	87/1	82/1	
rpsL	30S ribosomal protein S12					65/1
rpsM	30S ribosomal protein S13	31/2	57/3	41/2	60/1	76/1
rpsO	30S ribosomal protein S15		61/1			
rpsP	30S ribosomal protein S16	29/2			94/2	
rpoC	DNA-directed RNA polymerase subunit beta'	31/2	284/7	87/3	255/6	185/5
rsp_0003	Putative site-specific recombinase*	273/4	169/4	40/2	104/3	241/2
rsp_0111	NADH dehydrogenase subunit M		99/2	236/3	225/3	63/1
rsp_0251	Outer membrane efflux protein				128/2	
rsp_0290	**LhaA**	**71/1**	**317/2**	**348/3**	**479/2**	**527/4**
rsp_0332	Putative cellulose synthase					83/3
rsp_0356	Protein HflC		107/4	77/3		201/7
rsp_0446	Isocitrate dehydrogenase [NADP]					56/2
rsp_0465	Putative protease				71/1	
rsp_0668	OmpA/MotB family protein*	232/4	244/4	123/2	229/4	49/2
rsp_0841	Uncharacterized protein		81/1	53/2	93/3	
rsp_1033	OmpA family protein	67/2	443/3	289/3	294/3	174/3
rsp_1151	NADH-ubiquinone oxidoreductase			50/2	186/3	59/1
rsp_1200	Uncharacterized protein with SCP/PR1 domains*	91/2	150/3	137/2	196/3	
rsp_1239	Uncharacterized protein					24/2
rsp_1271	Periplasmic sensor signal transduction histidine kinase					53/1
rsp_1352	D-3-phosphoglycerate dehydrogenase		56/2			98/3
rsp_1415	Putative polysaccharide deacetylase	114/2	122/4		123/3	
rsp_1472	Uncharacterized protein		16/2			
rsp_1760	Uncharacterized protein				61/2	70/1
rsp_1762	Uncharacterized protein					175/3
rsp_2175	ABC transporter, ATPase subunit			53/2	134/3	
rsp_2698	Putative quinoprotein		112/2	52/3		
rsp_2812	ABC-type uncharacterized transport auxiliary component		85/1			
rsp_2903	Periplasmic chaperone for outer membrane proteins SurA		41/2			
rsp_2948	Indolepyruvate ferredoxin oxidoreductase			35/2	35/2	69/1
rsp_3229	Membrane-bound lytic murein transglycosylase B*	58/3	65/2	17/2	22/2	
rsp_3238	Uncharacterized protein		139/3	357/7	492/6	426/7

rsp_3361	Putative restriction endonuclease or methylase					55/1
rsp_4157	Radical SAM superfamily protein				54/1	45/2
rsp_4158	Generic methyltransferase		49/2			143/2
rsp_6001	Peptidase inhibitor I78 family*	141/1	166/2		71/1	87/1
rsp_6161	SH3 domain-containing protein*	90/2	102/2	76/3	161/3	
rsp_7517	Uncharacterized protein*	65/1	75/1		58/1	
secE	Protein translocase subunit SecE					52/1
tufA	Elongation factor Tu*	115/3	253/4	186/4	275/6	522/8
yajC	Protein translocase subunit ~~yajC~~					157/2
ybaU	Putative peptidyl-prolyl cis-trans isomerase				37/3	45/3

Membrane preparations from a mutant expressing N-terminal FLAG-LhaA were solubilised with 0.1 – 3.0% β-DDM and the clarified extracts applied to anti-FLAG resin (see *Experimental procedures*). The captured proteins, shown in alphabetical order, were identified via their tryptic peptide fragments by database searching as described in *Experimental procedures*. The database search results, in which the MOWSE scores represent the inverse of the probability that an observed match is a random event, were filtered according to the following criteria: (1) significance threshold $p < 0.05$, (2) significant peptide count ≥ 2 and (3) for proteins detected with 1 significant peptide, MOWSE score ≥ 50. Proteins shown with asterisks were also detected in the negative control. The false discovery rates (FDRs) for these searches were 1.90, 0.60, 0.40, 1.03 and 0.29% respectively.

Table S4. Proteins identified from a FLAG-PucC co-immunoprecipitation assay.

**Gene name**	**Protein name**	**MOWSE score/Significant peptide count**
atpA	ATP synthase subunit alpha*	146/6
atpD1	ATP synthase subunit beta 1	159/5
bamA	Outer membrane protein assembly factor BamA	119/2
bchE	Anaerobic Mg-protoporphyrin IX monomethyl ester cyclase	29/3
bchI	Magnesium-chelatase 38 kDa subunit	75/3
ftsH	ATP-dependent zinc metalloprotease FtsH	58/3
groL1	60 kDa chaperonin 1	28/2
metK	S-adenosylmethionine synthase	133/4
PSrp1	Ribosomal subunit interface protein Y	74/2
pucB	Light-harvesting protein B-800/850 beta chain	118/2
pucC	**PucC**	**810/4**
pufM	Reaction center protein M chain	117/2
puhA	Reaction center protein H chain*	17/2
rpoC	DNA-directed RNA polymerase subunit beta'	133/3
rplB	50S ribosomal protein L2	28/2
rplP	50S ribosomal protein L16	55/1
rpsB	30S ribosomal protein S2	83/3
rpsD	30S ribosomal protein S4*	29/2
rsp_0293	Putative photosynthetic complex assembly protein	71/1
rsp_1762	Uncharacterized protein	301/6
rsp_3238	Uncharacterized protein	110/3
tufA	Elongation factor Tu*	321/5

A membrane preparation from a mutant expressing N-terminal FLAG-PucC was solubilised with 3% β-DDM on the basis that, in the FLAG-LhaA experiment, this concentration produced the maximum number of identifications with the highest target protein score (see Table S3). The captured proteins, including the target protein PucC (shown in bold), were identified with the results filtered and shown as described in Table S3. Proteins shown with asterisks were also detected in the negative control. The FDR for this search was 0%.

Table S5. Proteins identified from a FLAG co-immunoprecipitation negative control.

Gene name	Protein name	MOWSE score/Significant peptide count
atpA	ATP synthase subunit alpha	80/4
comL	Outer membrane protein assembly factor BamD	27/2
exoP	Putative succinoglycan biosynthesis transport protein	293/8
glnA1	Glutamine synthetase	63/2
pucB	Light-harvesting protein B-800/850 beta chain	66/2
pucC	PucC	28/2
puhA	Reaction center protein H chain	26/2
rpsC	30S ribosomal protein S3	40/2
rpsD	30S ribosomal protein S4	64/2
rsp_0003	Putative site-specific recombinase	140/2
rsp_0243	Putative lipoprotein	51/2
rsp_0668	OmpA/MotB family protein	206/4
rsp_1200	Uncharacterized protein with SCP/PR1 domains	193/3
rsp_2847	Uncharacterized protein	42/2
rsp_3229	Membrane-bound lytic murein transglycosylase B	115/3
rsp_3565	Glycoside hydrolase, family 25	61/2
rsp_6001	Peptidase inhibitor I78 family	242/2
rsp_6161	SH3 domain-containing protein	157/3
rsp_7517	Uncharacterized protein	85/1
tufA	Elongation factor Tu	25/2

A membrane preparation from wild-type strain 2.4.1, expressing no FLAG-tagged proteins, was solubilised with 3% β-DDM and the clarified extract applied to anti-FLAG resin as per the FLAG-LhaA and -PucC co-immunoprecipitation method (see *Experimental procedures*). The captured proteins were identified with the results filtered and shown as described in Table S3. The FDR for this search was 1.74%.

Table S6. Proteins identified from FLAG-LhaA ICM and UPB CN-PAGE bands containing LhaA.

Gene name	Protein name	MOWSE score/Significant peptide count ICM Band 1	ICM Band 2	UPB Band 1
atpA	ATP synthase subunit alpha		54/1	
bchE	Anaerobic Mg-protoporphyrin IX monomethyl ester cyclase	43/4	126/4	
bchG	Bacteriochlorophyll synthase 33 kDa chain			92/2
bchP	Geranylgeranyl hydrogenase	131/2		190/4
ccmF	Cytochrome c maturation protein		132/2	
ccoN	Cbb3-type cytochrome oxidase CcoN subunit		61/1	
crtA	Spheroidene monooxygenase	99/4	793/10	73/1
crtI	Phytoene dehydrogenase	216/6		290/7
cycA	Cytochrome c_2		128/1	
fbcB	Cytochrome *b*	684/6		465/5
fbcC	Cytochrome c_1	81/3		65/2
ftsH	ATP-dependent zinc metalloprotease FtsH	59/1		329/4
groL1	60 kDa chaperonin 1	140/1	62/1	738/12
hemH	Ferrochelatase		123/2	
Int	Apolipoprotein N-acyltransferase	77/1		
lolE	ABC lipoprotein efflux transporter, inner membrane	79/1		
lpxB	Lipid-A-disaccharide synthase			81/3
mraY	Phospho-N-acetylmuramoyl-pentapeptide-transferase		54/1	
psd	Phosphatidylserine decarboxylase proenzyme	628/8		492/8
pgsA	Phosphatidylglycerophosphate synthase	53/1		82/1
pufL	Reaction center protein L chain		237/4	
pufM	Reaction center protein M chain		898/3	
puhA	Reaction center protein H chain		1133/18	
qxtA	Cytochrome bd_1 ubiquinol oxidase subunit 1		167/3	
rplF	50S ribosomal protein L6			121/4
rplK	50S ribosomal protein L11			148/1
rplW	50S ribosomal protein L23			130/1
rpmB	50S ribosomal protein L28			104/2
rpsB	30S ribosomal protein S2			472/8
rsp_0111	NADH dehydrogenase subunit M	338/3		161/2
rsp_0290	**LhaA**	**130/3**	**EIC**[1]	**191/3**
rsp_0293	Putative photosynthetic complex assembly protein	143/1		184/1
rsp_0477	Putative hydrolase of the alpha/beta-hydrolase fold protein	211/4		234/5
rsp_0668	OmpA/MotB family protein			325/4
rsp_0963	Inner membrane protein	57/2		47/2
rsp_1033	OmpA family protein	322/4		325/4
rsp_1151	NADH-ubiquinone oxidoreductase	68/1		81/3
rsp_1200	Uncharacterized protein with SCP/PR1 domains			108/2
rsp_1232	UPF0093 membrane protein RHOS4_28450			79/1
rsp_1467	Alkane 1-monooxygenase	448/8		376/6
rsp_1674	Signal peptidase I	64/2		
rsp_1760	Uncharacterized protein	392/4	105/1	53/1
rsp_1762	Uncharacterized protein	163/3		61/1
rsp_1812	Amino acid/amide ABC transporter substrate-binding protein, HAAT family			406/6
rsp_2465	Putative uncharacterized protein			
rsp_2673	Quinoprotein glucose dehydrogenase	451/8		175/5
rsp_2718	Putative outer membrane protein	531/5	222/4	653/5
rsp_2803	Multidrug/cation efflux pump, RND superfamily	125/3		

rsp_2845	Putative penicillin acylase	191/6		454/11
rsp_2847	Uncharacterized protein			70/3
rsp_2908	Uncharacterized protein			54/1
rsp_3332	Putative transmembrane protein	69/1		
rsp_3588	Putative integral membrane protein	384/4	178/2	282/2
rsp_3719	Polysaccharide export transporter, PST family			56/1
secD	Protein translocase subunit SecD	29/2		
secE	Protein translocase subunit SecE			57/1
secG	Protein translocase subunit SecG			51/1
ssPA	Salt-stress induced outer membrane protein		108/2	
tatA	Sec-independent protein translocase protein TatA	83/1		54/1
yajC	Protein translocase subunit ~~yajC~~		469/3	154/2
ybaU	Putative peptidyl-prolyl cis-trans isomerase	68/2	46/2	
yidC	Membrane protein insertase YidC		119/4	383/8

ICM and UPB preparations from a mutant expressing N-terminal FLAG-LhaA were analysed by CN-PAGE and bands containing LhaA, identified on immunoblots probed with anti-FLAG antibody, were excised and subjected to in-gel digestion with trypsin (see *Experimental procedures*). The proteins contained in the bands were identified with the results filtered and shown as described in Table S3. The FDRs for these searches were 2.20, 2.20 and 2.90% respectively.

[1]LhaA was identified from an extracted ion chromatogram (EIC) of the MS spectra, not in the database search.
